# Transcriptome analysis and identification of genes associated with ω-3 fatty acid biosynthesis in *Perilla frutescens* (L.) var. *frutescens*

**DOI:** 10.1186/s12864-016-2805-0

**Published:** 2016-06-24

**Authors:** Hyun Uk Kim, Kyeong-Ryeol Lee, Donghwan Shim, Jeong Hee Lee, Grace Q. Chen, Seongbin Hwang

**Affiliations:** Department of Bioindustry and Bioresource Engineering, Plant Engineering Research Institute, Sejong University, Seoul, 05006 Republic of Korea; Department of Agricultural Biotechnology, National Institute of Agricultural Science, Rural Development Administration, Jeonju, 54874 Republic of Korea; Department of Forest Genetic Resources, National Institute of Forest Science, Suwon, 16631 Republic of Korea; SEEDERS Inc, Daejeon, 34015 Republic of Korea; U.S. Department of Agriculture, Western Regional Research Center, Agricultural Research Service, 800 Buchanan Street, Albany, CA 94710 USA

**Keywords:** *Perilla frutescens*, Seed, ω-3 fatty acid, α-linolenic acid, Triacylglycerol, Transcriptome

## Abstract

**Background:**

Perilla (*Perilla frutescens* (L.) var *frutescens*) produces high levels of α-linolenic acid (ALA), a ω-3 fatty acid important to health and development. To uncover key genes involved in fatty acid (FA) and triacylglycerol (TAG) synthesis in perilla, we conducted deep sequencing of cDNAs from developing seeds and leaves for understanding the mechanism underlying ALA and seed TAG biosynthesis.

**Results:**

Perilla cultivar Dayudeulkkae contains 66.0 and 56.2 % ALA in seeds and leaves, respectively. Using Illumina HiSeq 2000, we have generated a total of 392 megabases of raw sequences from four mRNA samples of seeds at different developmental stages and one mature leaf sample of Dayudeulkkae. *De novo* assembly of these sequences revealed 54,079 unique transcripts, of which 32,237 belong to previously annotated genes. Among the annotated genes, 66.5 % (21,429 out of 32,237) showed highest sequences homology with the genes from *Mimulus guttatus*, a species placed under the same Lamiales order as perilla. Using Arabidopsis acyl-lipid genes as queries, we searched the transcriptome and identified 540 unique perilla genes involved in all known pathways of acyl-lipid metabolism. We characterized the expression profiles of 43 genes involved in FA and TAG synthesis using quantitative PCR. Key genes were identified through sequence and gene expression analyses.

**Conclusions:**

This work is the first report on building transcriptomes from perilla seeds. The work also provides the first comprehensive expression profiles for genes involved in seed oil biosynthesis. Bioinformatic analysis indicated that our sequence collection represented a major transcriptomic resource for perilla that added valuable genetic information in order Lamiales. Our results provide critical information not only for studies of the mechanisms involved in ALA synthesis, but also for biotechnological production of ALA in other oilseeds.

**Electronic supplementary material:**

The online version of this article (doi:10.1186/s12864-016-2805-0) contains supplementary material, which is available to authorized users.

## Background

*Perilla frutescens*, commonly called perilla, is a cultivated crop of the mint family Lamiaceae. Two distinct varieties, *P. frutescens* var. *frutescens,* the oilseed crop for source of perilla oil, and *P. frutescens* var. *crisp* for the aromatic leafy herb, are cultivated in East Asia countries mainly in Korea, Japan and China [1]. *P. frutescens* var. *frutescens*, hereafter called perilla, contains 35–45 % triacylglycerol (TAG) in seeds. It is a rich source of poly unsaturated fatty acids (FA) showing 54–64 % of ω-3 FA (ɑ-linolenic acid, ALA or 18:3) and 14 % ω-6 FA (linoleic acid, LA or 18:2) [2]. Major oil seed crops (e.g., soybean, rapeseed, maize, peanut and sunflower) have relatively low ω-3 FA content (below 10 % in total FAs) in seed TAGs. The ω-3 and ω-6 FAs confer various health benefits for human [3]. The recommended ω-6/ω-3 FA ratio in human diet is 2:1 or lower [4, 5]. However, a typical human diet has high ω-6/ω-3 FA ratio (approximately 15:1) which is considered as a major contributor to cardiovascular diseases [5]. Perilla seed oils have an approximately 0.2:1 ratio of ω-6/ω-3 FAs. This extremely low ratio of ω-6/ω-3 FAs makes perilla a desirable dietary source of vegetable oils [2]. Perilla oil also has many industrial uses, such as for drying oil in paint, varnish and ink manufacturing or as a substitute for linseed oil [6]. Perilla seed cakes are used as animals and birds feed.

Most research for perilla has been focused on identification of metabolites and their biological activities for human health [7, 8]. Some of the genes involved in the biosynthesis of anthocyanins, flavones and monoterpenoids have been studied [9, 10]. Recent reports on the generation of transcriptome using high-throughput sequencing were primarily for identification of genes for anthocyanin pathways associated with red or green leaf varieties of perilla [11, 12]. In contrast, studies on the molecular basis of seed FA and TAG synthesis in perilla have been limited. A seed-specific omega-3 fatty acid desaturase cDNA has been cloned [13] and characterized in perilla [14]. An oleosin promoter from perilla was found to have a seed-specific activity in transgenic Arabidopsis [15]. Besides perilla, flax (*Linum usitatissimum*), sacha inchi (*Plukenetia volubilis L.*), and chai (*Salvia hispanica L*., a member of mint family *Lamiacease*) also contain high percentage of ALA in seed oil [14]. Seed transcriptome data of Chai [16] and sacha inchi (*Plukenetia volubilis L.*) [17] have been published, but a few genes contributing to the accumulation of ω-3 FA have been characterized for their expression profiles during seed development.

In this study, we adopted Illumina HiSeq 2000 platform aiming at analyzing the seed transcriptome of perilla. A leaf transcriptome was also included which allows comparison and detection of differentially expressed gene (DEG) in developing seeds of perilla. We have identified 54,079 unique transcripts from a total of 392 mega-base raw sequences, including transcripts for the majority of enzymes involved in lipid biosynthesis and metabolism. We further characterize the expression profiles of 43 key genes involved in FA and TAG in developing seeds and leaf using quantitative PCR (qPCR) assays. To our knowledge this work describes the first seed transcriptome of perilla, and also the first spatial and temporal expression patterns of all known key genes for FA and TAG synthesis in perilla. Our results provide important information for understanding the mechanisms involved in ALA accumulation in perilla.

## Results and discussion

### Fatty acid profile in developing seeds and leaf

**Fig. 1:**
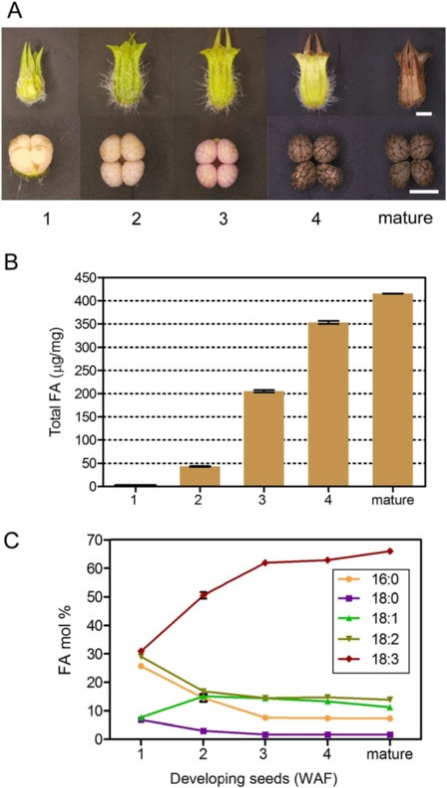
Seed development and fatty acids composition of *Perilla frutescens* (L.) var. *frutescens.* **a** Photographs of seeds from 1 to 4 weeks after flowering (WAF) and mature seeds. Scale bar indicate 0.5 cm. **b** Change of total fatty acid content during seed development. **c** Fatty acid composition during seed development. Biological triplicates were averaged. Bars indicate the standard error (SE) of the mean

To investigate the relationships between FA profile and gene expression, we studied seed development in perilla. We harvested developing seeds 1–4 weeks after flowering (WAF) during seed development and found that seeds matured at 5 WAF (Fig. 1a). These harvested seed samples were analyzed for their FA content and composition by gas chromatograph (GC). During seed development, total FAs were measured at a very low level of 2.8 μg.mg^−1^ in seeds at 1 WAF and increased steadily to 43 μg.mg^−1^ at 2 WAF, 205 μg.mg^−1^ at 3 WAF and 353 μg.mg^−1^ at 4 WAF (Fig. 1b). We measured an average of total FA at 415 μg.mg^−1^ in mature seeds (5 WAF and older), which is about 40 % of seed dry weight. Mature seeds at 5 WAF contained 66 % of ALA, 13.8 % of LA, 11.2 % of oleic acid (18:1 Δ9), 1.7 % of stearic acid (18:0), and 7.3 % of palmitic acid (16:0) (Fig. 1c). ALA was also found to be a predominant FA in young seeds, showing 30.9 and 50.5 % at 1 and 2 WAF, respectively (Fig. 1c). After 2 WAF, ALA level gradually reached a plateau level of 66 %. Oleic acid was 7.7 % in seeds at 1 WAF and increased to 15.1 % at 2 WAF and maintained similar levels thereafter to the maturation stage. The remaining three FAs, palmitic acid, stearic acid and LA, had slight declining accumulation patterns during seed development. These FAs had levels at 25.7, 6.8 and 29 %, respectively at 1 WAF, then declined to about half of their beginning levels at 2 WAF, and maintained the levels throughout the remaining stages of seed development. In a leaf sample, we also detected a high level of ALA at 56.2 %, similar to that of mature seeds (Additional file 1: Table S1). Besides ALA, there are six FAs in leaves, represented at 14.9 % for LA, 3.3 % for oleic acid, 2 % for stearic acid, 4.3 % for hexadecatrienoic acid (16:3), 2.2 % for palmitoleic acid (16:1), and 17.2 % for palmitic acid (Additional file 1: Table S1). Our observed spatial and temporal patterns of FAs in developing seeds and leaf tissues were similar to described [14].

### Transcriptome sequencing of perilla and *de novo* assembly

RNA samples from four different stages of developing seeds (1–4 WAF) and leaf were sequenced using Illumina Hiseq2000 system, which generated total 392,479,798 reads. After trimming the adapter and low quality reads and removing those shorter than 25 bp, a total 372,171,322 high quality reads were obtained from combined four different stages of developing seeds and one leaf libraries (Table 1). These reads were assembled into 191,545 contigs (or transcripts) (N50 = 1900 bp) and 80.7 % of them (154,621 transcripts) were annotated (Additional file 1: Table S2). Using a sequence similarity cutoff of 95 %, the assembled sequences were clustered into 54,079 unique transcripts, with an average length of 871 bp and total size of 47.1 Mb (Additional file 1: Table S2). Transcripts and unique transcripts were searched against the Phytozome database (http://phytozome.jgi.doe.gov) using BLASTx with E-value cut-off of 1E-10. The search resulted 154,621 transcripts and 32,237 unique transcripts (Additional file 1: Table S2). These sequences had at least one match to known protein sequences in 39 plant species (Additional file 1: Tables S2, S3 and S4). Analysis of length distribution in assembled transcripts indicated that the transcripts varied between 1 to >3601 bp (Additional file 2: Figure S1). Transcripts with 301–600 bp were the most abundant among the assembled transcripts and unique transcripts (Additional file 2: Figure S1). Two transcriptome data from leaves of perilla variety, *P. frutescens* var. *frutescens* Britt (Accession name:PF98095) [11] and *P. frutescens* var. *crispa* [12], were yielded 48,009 and 54,500 transcripts with average length of 873, and 824 bp, respectively. Our transcriptome assembly showed similar number of 54,079 unique transcripts. However, our N50 unique transcript size of our perilla was 1401, which is higher than 904 bp for *P. frutescens* var. *frutescens* Britt [11] and 1349 bp for *P. frutescens* var. *crispa* [12].

**Table 1:** Summary of sequencing data of *P. frutescens* seeds and leaf transcriptomes

	Seed				Leaf	Total
	1 week	2 weeks	3 weeks	4 weeks		
Total number of raw reads	59,619,730	64,434,520	98,130,006	67,528,198	102,767,344	392,479,798
Total number of clean reads	57,081,328	61,445,940	92,851,684	63,305,772	97,486,598	372,171,322
^a^Trimmed/raw (%)	95.7	95.4	94.6	93.7	94.9	94.8

^a^Trimmed/raw: Total trimmed read/total raw read

### Functional annotation of perilla transcriptome

We validated and annotated the unique transcripts with BLASTx homology search in Phytozome database. Among total 32,237 annotated unique transcripts, 21,429 transcripts (66.5 %) are highly matched with proteins from *Mimulus guttatus* (Monkey flower), followed by 1709 (5.3 %), 1431 (4.4 %), and 977 (3.0 %) transcripts matched with proteins from *Solanum tuberosum*, *Solanum lycopericum* and *Vitis vinifera*, respectively. The remaining 6691 (21 %) transcripts matched protein sequences from 37 plant species (Additional file 2: Figure S2). It is not a surprise that most perilla transcripts have high sequence homology to *M. guttatus* [18], as both species are under the same Lamiales order. The results allow the translation of genomics and genetics research findings between *M. guttatus* and perilla.

### Analysis of differentially expressed genes (DEG) in perilla developing seeds

To examined the difference in gene expression between seeds and leaves, we performed a DEG analysis using bowtie2 (v2.1.0) [19]. The up- or down-regulated genes were determined by comparison with the level of corresponding genes in leaf. The number of transcripts with > 2-fold change with a false discovery rate (FDR) < 0.01 was presented in Additional file 2: Figure S3. In developing seeds at 1, 2 and 3 WAF, the numbers of up-regulated genes were about 28–48 % less than that of down-regulated genes, showing 1184, 1052 and 1032, respectively; whereas the number of down-regulated genes presented at 1640, 2027 and 2151, respectively (Additional file 2: Figure S3). When seeds reached to maturation at 4 WAF, the number of up- and down-regulated genes had almost identical numbers, 2059 or 2058 (Additional file 2: Figure S3). As we can see, the numbers (1032-1184 counts) of up-regulated genes were similar in seeds at the first three stages (1–3 WAF), and increased to 2-fold (2,059 counts) in 4 WAF. Whereas the numbers of down-regulated genes (2027–2059 counts) were similar in seeds at late three stages (2–4 WAF). The DEG detected in this study provides a global view of seed transcriptome which is important for further investigation of the molecular basis of seed development not only in perilla, but also in other oilseeds.

### Clustering of DEGs

Hierarchical clustering was performed with the 6012 DEGs using Another Multidimensional Analysis Package (AMAP) library in R [20] to examine the similarity and diversity of expression profiles. Similarity of expression pattern of genes was estimated with pearson’s correlation. The results are displayed by Java Treeview (Additional file 2: Figure S4A). The normalized values are represented by different colors, with red representing positive values and green representing negative values. The analysis resulted in twelve clusters (Additional file 2: Figure S4B). Cluster 1 (374 DEGs) and 6 (602) had a similar declining pattern showing a higher level in seeds at 1 WAF, and decreased levels during the rest stages of the development (Additional file 2: Figure S4B). These DEGs may be important for early seed development. DEGs in Cluster 2 (1851) were down-regulated in seeds at all stages indicating that these genes were involved in cell metabolism in leaf. In contrast, DEGs in Cluster 3, 4 and 5 were all up-regulated with slightly different trends showing concave/flat, concave/rise and convex/flat, respectively. These DEGs were likely seed specific genes. Genes in Cluster 7–12 were less differentially expressed between leaf and seeds (Additional file 2: Figure S4B). Cluster 7 (51) had a convex/flat pattern with slightly higher expression levels in seeds at early (1 WAF) and late (4 WAF) stages. Cluster 8 (106) and 10 (118) had similar concave/flat expression patterns and both peaked in 2 WAF seeds. Cluster 9 (478) and 11 (131) were both flat/rise and peaked at 4 WAF. Cluster 12 (37) showed concaved/rise with a peaked expression at 3 WAF. The above variable temporal patterns indicate that multiple mechanisms were involved in regulating gene expression during perilla seed development. Similar temporal patterns of DEGs were also observed in other oilseeds [21–23].

### Analysis of seed abundant DEGs in Cluster 3, 4, 5 and 10

Gene Ontology (GO) analysis was further used to classify functions of transcripts in cluster 3, 4, 5, 10 DEG. Using DAVID (http://david.abcc.ncifcrf.gov/tools.jsp) based on the Arabidopsis Information Resource Gene Ontology classification [24], a total of 2870 DEGs were categorized into 43 functional groups under main GO terms: cellular component, molecular function and biological process. DEGs in all four Cluster 3, 4, 5 and 10 showed similar functions. In the biological process, most transcripts were assigned to “nitrogen compound metabolic process (264 counts,)”, followed by “cellular metabolic process (235)”, “biosynthetic process (221) and “primary metabolic process (141)”. In the cellular components category, the majority of transcripts were associated with the terms “cell periphery (264)”, followed by “protein complex (57)”, and “organelle membrane (31)”. In the molecular function group, the majority of transcripts were related to the terms “ion binding (299)”, “transferase activity (281)” “hydrolase activity (249)”, “oxidoreductase activity (180)” and “transmembrane transporter activity (81)” (Additional file 1: Table S5). Additional file 1: Table S6 lists the top 50 DEGs from Cluster 3, 4, 5 and 10. Among them, the most abundant genes are seed storage proteins (cruciferin, cupins and late embryogenesis abundant (LEA) proteins) and lipids biosynthesis genes, including oleosins, hydroxysteroid dehydrogenase I for TAG biogenesis, acyl carrier protein and *FAB2, FAD7/8* and *FAD2* for FA synthesis.

### Analysis of acyl-lipid genes in developing seeds

The most comprehensive database of plant acyl-lipid genes and pathways have been constructed for Arabidopsis (http://arabidopsisacyllipids.plantbiology.msu.edu/pathways/pathways) [25]. To identify acyl-lipid genes involved in seed oil biosynthesis in perilla, we searched perilla assembled genes using Arabidopsis acyl-lipid genes as queries. Among 975 queries, a total of 540 unique transcripts were identified from perilla transcriptome (Additional file 1: Table S7), which is about 55 % matchup. A similar result (57 % match up) was obtained when searching lesquerella (*Physaria fendleri*) transcriptome using Arabidopsis acyl-lipid genes as queries [26]. Considering lesquerella and Arabidopsis both belong to the same Brassicaceae, whereas perilla and Arabidopsis are from different order, our results indicate that acyl-lipid genes are conserved among different plant species. Furtherly, we have focused on 43 major genes whose functions are likely responsible for FA and TAG biosynthesis based on our knowledge from model Arabidopsis (Additional file 1: Table S8). Deduced amino acid sequences of perilla genes had varied sequence identities with those of Arabidopsis genes, showing a relatively higher range of 74–92 % for FA biosynthesis than 41–87 % for TAG assembly. Perilla oleosins involved in oil-body formation showed 51–69 % identity compared with those of Arabidopsis. Our data indicate that between perilla and Arabidopsis, genes for FA biosynthesis in plastid are more conserved than those for TAG assembly in ER. The high content of ALA in perilla seed TAG (Fig. 1b and c) is probably resulted from some the genes in ER modified through evolution and become favorable for generating ALA in seed oils.

### Genes for FA biosynthesis in plastids

**Fig. 2:**
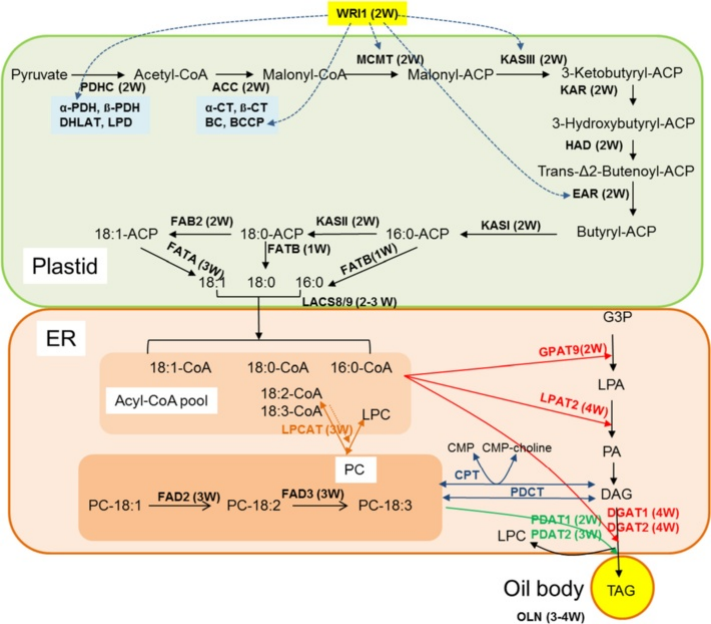
Predicted FA and TAG biosynthetic pathways in perilla *s*eeds. Numbers after the genes (#W) indicate the highest WAF of its expression in developing seeds. Inside of bright green rectangle presents FA biosynthesis and fatty acid export from plastid. Inside of bright red rectangle presents glycerolipids biosynthesis for TAG formation in ER. Yellow circle indicate TAG in oil body. WRI1 transcription factor regulates *ɑ-PDH, BCCP, MCMT, KASIII* and *EAR* genes in FA biosynthesis. Red rectangles inside of ER represent acyl-CoA pools and reaction of desaturation by FAD2 and FAD3 in PC, respectively. Acyl-CoA dependent Kennedy pathway is indicated with red arrow. PC-mediated TAG synthesis pathways are indicated with green (by PDAT), blue (by PDCT and CPT) and orange (by LPCAT). Abbreviations: WRI1, wrinkled1; PDHC, plastidial pyruvate dehydrogenase complex; PDH, pyruvate dehydrogenase; DHLAT, dihydrolipoly acyltransferase; LPD, dihydrolipoamide dehydrogenase; ACC, acetyl-CoA carboxylase; CT, carboxyltransferase; BC, biotin carboxylase; BCCP, biotin carboxyl carrier protein; CoA, coenzyme A; ACP, acyl carrier protein; MCMT, malonly-CoA ACP transferase; KAS, ketoacyl-ACP synthase; KAR, 3-ketoacyl-ACP reductase; HAD, 3-hydroxyacyl-ACP dyhydratase; EAR, 2-enoyl-ACP reductase; FAB2, fatty acid biosynthesis2; FATA, acyl-ACP thioesterase A; FATB, acyl-ACP thioesterase B; LACS, long-chain acyl-CoA synthase; FAD2, Δ12 oleic acid desaturase; FAD3, Δ15 (ω-3) linoleic acid desaturase; GPAT9, glycerol 3-phosphate acyltransferase 9; LPAT2, lysophosphatidic acid acyltransferase 2; LPCAT, lysophosphatidylcholine acyltransferase; DGAT, diacylglycerol acyltransferase; PDAT, phospholipid: diacylglycerol acyltransferase; CPT, CDP-choline:DAG cholinephosphotransferase; PDCT, phosphatidylcholinediacylglycerol cholinephosphotransferase; G3P, glycerol-3-phosphate; LPA, lysophosphatidic acid; PA, phosphatidic acid; LPC, lysophosphatidylcholine; PC, phosphatidylcholine; DAG, diacylglycerol; TAG, triacylglycerol; OLN, oleosin

**Fig. 3:**
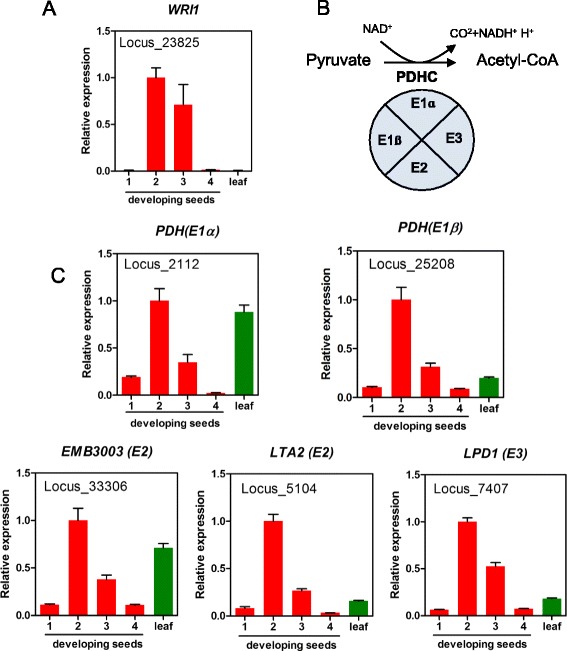
Synthesis of acetyl-CoA from pyruvate in plastids. **a** Expression of WRINKLED1 (WRI1). **b** Pyruvate dehydeogenase complex (PDHC) reaction. **c** Expression of five subunits genes of PDHC. Abbreviations are described as in Fig. 2

Knowledge of genes and pathways involved in seed oil biosynthesis has been extensively studied. FAs are synthesized in plastid and then exported to cytosol to be activated to acyl-Coenzyme A (CoA) for TAG assembly in ER [25, 27, 28]. Key genes and pathways involved in *de novo* FA biosynthesis in perilla plastids are proposed (Fig. 2). Acetyl-CoA generated from pyruvate by the plastidial pyruvate dehydrogenase complex (PDHC) is used as a starting substrate for FA synthesis (Figs. 2 and 3b). The PDHC is a large multienzyme containing three components: E1 (pyruvate dehydrogenase or PDH, composed of E1ɑ and E1ß subunits), E2 (dihydrolipoyl acyltransferase or DHLAT), and E3 (dihydrolipoamide dehydrogenase or LPD) [29]. In perilla transcriptomes, we have identified five subunit genes for PDHC: *PDH (E1ɑ), PDH (E1ß), EMB3003 (E2), LTA2(E2)* and *LPD1 (E3)* (Additional file 1: Table S8). Spatial and temporal analysis of gene expression of these five subunits indicated that they all expressed in leaf and seeds; during seed development, they all had similar bell-shaped patterns with peaks at 2 WAF (Fig. 3c). The results suggest that genes encoding subunit of PDHC are coordinated regulated for synthesizing acetyl-CoA in seed and leaf (Fig. 3).

**Fig. 4:**
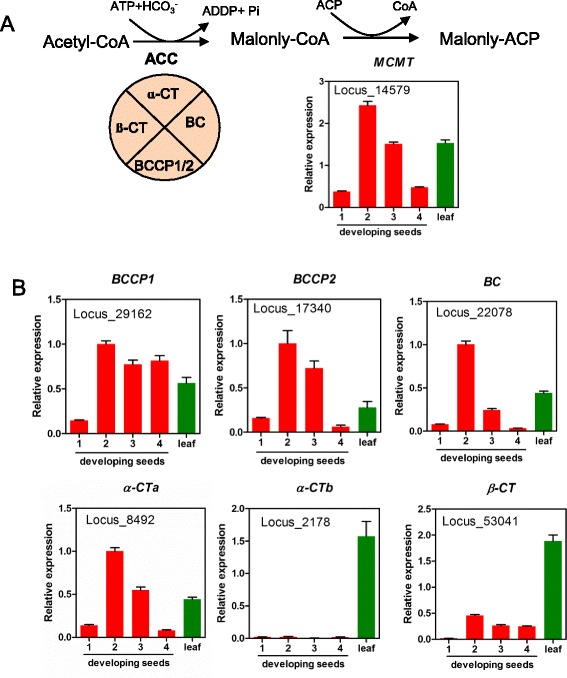
Synthesis of malonly-ACP from acetyl-CoA. **a** Malonyl-ACP synthesis from acetyl-CoA by heteromeric acetyl-CoA carboxylase (Het ACC) and malonyl-CoA:ACP malonyltransferase (MCMT). **b** Expression of subunits genes of Het ACC complex. Abbreviations are described as in Fig. 2

Once acetyl-CoA is synthesized, a heteromeric complex enzyme, acetyl-CoA carboxylase (ACC) catalyzes acetyl-CoA to form malonyl-CoA (Figs. 2 and 4a) [30]. A malonyl-CoA ACP transferase (MCMT) then further converts malonly-CoA to malonly-ACP. Perilla MCMT ortholog (Locus_14579) was identified showing 86 % homology with Arabidopsis MCMT (At2g30200) and it is expressed high in 2 WAF developing seeds (Fig. 8a). ACC is composed of 4 subunits, 3 of them, biotin carboxyl-carrier protein (BCCP), biotic carboxylase (BC) and alpha-carboxyltransferase (α-CT) are encoded from nuclear genome; and beta-carboxyltransferase (β-CT) is encoded in plastid genome [31]. Six subunit genes were identified from perilla seeds and leaf transcriptomes (Additional file 1: Table S8). They are isoforms of *BCCP1* and *BCCP2*, *BC*, isoforms of *α-CTa* and *α-CTb*, and *β-CT*. Spatial and temporal analysis of gene expression revealed that all of them were expressed significantly in leaf. During seed development, *BCCP1* (Locus_29162) had flat-rise from 2 to 4 WAF, and *BCCP2* (Locus_17340) had a bell-shaped pattern with high levels at 2 and 3 WAF. The *BC* also had a bell-shaped pattern, but with a high level only at 2 WAF. Isoforms *α-CTa* (Locus_8492) had a bell-shaped pattern similar to that of *BCCP2*. However *α-CTb* (Locus_2178) did not expressed in developing seeds. The *β-CT* (Locus_53041) was expressed moderately from 2 to 4 WAF (Fig. 4b). Thus, with exception of *α-CTb,* the other 5 genes *α-CTa, β-CT, BC* and *BCCP1* or *BCCP2* may coordinately work together in seeds.

**Fig. 5:**
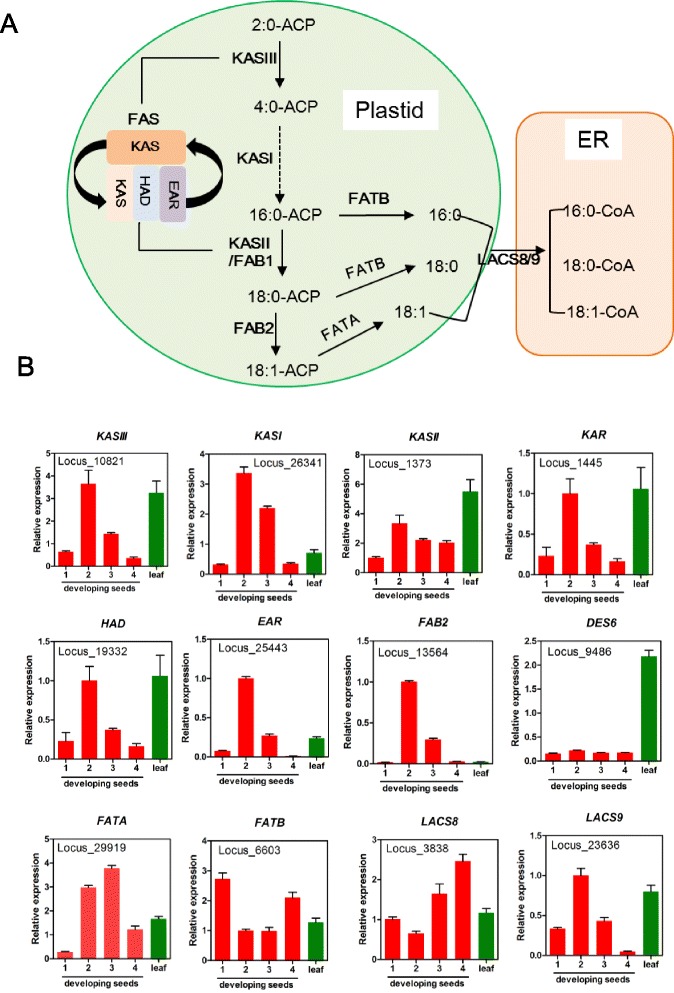
Synthesis of FAs and acyl-CoAs. **a** Pathway of synthesis of FA and acyl-CoA **b** Expression of genes for FA and acyl-CoA synthesis. Abbreviations are described as in Fig. 2

FA elongation is conducted by an acyl-chain specific condensing enzyme subunit, 3-keto-acyl-ACP synthase (KAS). and 3 common components, 3-ketoacyl-ACP reductase (KAR), 3-hydroxylacyl-ACP dehydratase (HAD) and 2-enoyl-ACP reductase (EAR) (Fig. 5a) [32]. KAS III, I and II specifically catalyze the reaction of acyl-chain elongation for 2:0-ACP to 4:0-ACP, 4:0-ACP to 16:0-ACP and 16:0-ACP to 18:0-ACP, respectively. Based on the sequence homology with Arabidopsis genes, we have identified perilla orthologs for all these subunits (Additional file 1: Table S8). Three KAS isoforms and three component of fatty acid synthase (FAS) showed similar temporal expression patterns during seed development (Fig. 5b). WRINKELD1 (WRI1) is a transcription factor for regulating some of the genes associated to FA biosynthesis in plastids. Arabidopsis *WRI1* regulates *α-PDH, BCCP2, MCMT, KASIII* and *EAR* genes in FA biosynthesis in seed development (Fig. 2) [33]. Perilla *WRI1* ortholog (Locus_23825) showed 82 % identity with that of Arabidopsis and it had a seed-specific expression pattern with a peak at 2 WAF (Fig. 3a). Overexpression of Brassica and maize WRI increased in 10–40 % of seed oils in transgenic plants [34, 35]. Perilla WRI1 could be used for engineering oilseeds for increased FA production in plastids which would provide increased FA supply for TAG assembly.

Stearoyl-ACP desaturase (SAD) catalyzes 18:0-ACP to 18:1-ACP in plastid (Fig. 2). Arabidopsis has seven SAD family genes included *FAB2* (At2g43710), and FAB2 plays a major role in producing 18:1 [36]. Perilla ortholog (Locus_13564) of Arabidopsis *FAB2* was detected in the seed transcriptome, and a homologue of At1g43710, *DES6* (Locus_9486), was detected in the leaf transcriptome. Detailed analysis of gene expression confirmed that indeed perilla *FAB2* and *DES6* were differentially expressed in seeds and leaf, respectively (Fig. 5b). 16:0-ACP, 18:0-ACP and 18:1-ACP are hydrolyzed to the acyl moiety from ACP by two fatty acid thioesterases. FATA and FATB are specific to 18:1-ACP and 16:0 or 18:0-ACPs, respectively. Two fatty acid thioesteases *FATA* (orthologous Locus_29919 of At3g25110) and *FATB* (orthologous Locus_6603 of At1g08510) were both detected in perilla seeds and leaf. However, the temporal expression of *FATA* and *FATB* were complementary to each other, showing a bell-shaped pattern with high levels at 2 and 3 WAF for *FATA* and inverted bell curve with high levels at 1 and 4 WAF for *FATB* (Fig. 5b). The higher expression of *FATA* at 2–3 WAF would suggest more 18:1 were terminated and released to ER, coinciding with the stages when seeds underwent rapid TAG synthesis. The highest transcript level of *FATB* detected in seeds at 1 WAF would suggest a swift demand of 16:0 and 18:0 for membrane biosynthesis at the onset of seed development, consisting with the higher levels of 16:0 and 18:0 detected in seeds at 1 WAF (Fig. 1c) [37]. Long chain acyl-CoA synthase (LACS) is located membrane of plastid outer envelope and/or ER and catalyzes free fatty acid to add Coenzyme A (CoA) for producing fatty acyl-CoA. Two perilla *LACSs*, *LACS8* (Locus_3838 ortholog of At2g04350) and *LACS9* (Locus_23636 ortholog of At1g77590), were identified. Expression of the *LACS9* exhibited a bell-shaped pattern with a maximum level at 2 WAF (Fig. 5b), which may associate with the increased demand of FA-CoA formation in cytosol [38] when developing seeds entering rapid growth phase. LACS9 was localized in plastid outer envelope [38]. For the *LACS8*, more transcripts were detected in seeds at 3–4 WAF than 1–2 WAF (Fig. 5b), therefore, the ER-localized LACS8 might be involved in TAG synthesis [39].

#### Desaturases associated with ER

**Fig. 6:**
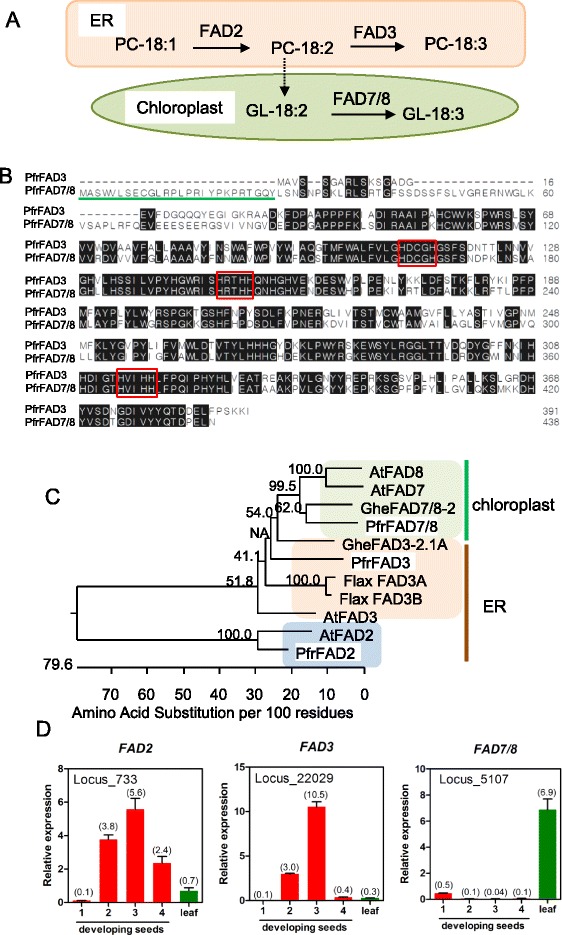
Polyunsaturated FA synthesis. **a** Polyunsaturated fatty acid synthesis pathway in ER and chloroplast. GL, glycerolipids. **b** Amino acid alignment of perilla (Pfr) FAD3 and FAD7/8. Red rectangles indicate His box, green line indicates a chloroplast transit peptide. **c** Phylogenetic analysis of plant FAD2, FAD3 and FAD8 desaturases. **d** Expression of *FAD2*, *FAD3* and *FAD7/8*. Abbreviations are described as in Fig. 2

ER contains two desaturases, FAD2 and FAD3. These enzymes catalyze desaturation of FAs attached to PC from PC-18:1 to PC-18:2 (FAD2) and from PC-18:2 to PC-18:3 (FAD3) [40–42] (Fig. 6a). During the search of perilla transcriptomes, we uncovered three loci, Locus_773, Locus_22029 and Locus_5107 encoding desaturases. Results of sequence alignment showed Locus_773 having 79 % of identity with the first plant *FAD2* from Arabidopsis (*AtFAD2,* Additional file 2: Figure S5A) [41]. Further phylogenetic analysis of 32 plant FAD2s revealed that the perilla sequence fell in a clade belong to constitutive type FAD2s (Additional file 2: Figure S5B). We designated this sequence encoding perilla FAD2 (PfrFAD2, Fig. 6c, Genbank ID: KP070823). During seed development, *PfrFAD2* expressed at a low level at 1 WFA, but it elevated to 33.8-, 50.0- and 21.2-fold at 2, 3 and 4 WFA, respectively (Fig. 6d). In leaf, *PfrFAD2* expressed at a significantly level showing 6.3-fold higher than that of seeds at 1WAF (Fig. 6d). In some plant species, such as Brassica and Camelina, additional *FAD2s* exist as seed-type isoforms [43, 44]. Without whole genome sequences, we cannot exclude the possibility of perilla having a seed-type FAD2 isoform. Nevertheless, our spatial and temporal gene expression data indicate that *PfrFAD2* plays an essential role in generating PC-18:2 by desaturation of PC-18:1 in both leaf and seed. We found Locus_22029 and Locus_5107 sharing the same sequence as published FAD3 (Genbank ID: KP070824) and FAD7/8 (Genbank ID: U59477.1), thus designated them to encode PfrFAD3 and PfrFAD7/8, respectively (Fig. 6b). Sequence alignments showed three His box conserved domains (HDCGH, HRTHH and HVIHH) among *PfrFAD3*, *PfrFAD7/8*, *AtFAD3*, *AtFAD7* and *AtFAD8* (Additional file 2: Figure S6) [45]. Besides, PfrFAD3 showed 70 % protein sequence identity with AtFAD3. PfrFAD7/8 showed 75 and 76 % identity with AtFAD7 and AtFAD8, respectively (Additional file 2: Figure S6). Between PfrFAD3 and PfrFAD7/8, a 68 % identity in protein sequences was detected (Fig. 6b). Phylogenetic analysis of desaturase genes among perilla, Arabidopsis [42], cotton [45] and flax [46] indicated that PfrFAD3 fell to ER-localized clade, whereas PfrFAD7/8 fell to chloroplast-localized clade (Fig. 6c). Results of our gene expression analysis indicated that expression of *PfrFAD3* were highly elevated in seeds at 2–3 WAF, showing 45.2- and 160.0-fold induction compared with the level in seeds at 1 WFA (Fig. 6d). A previous Northern analysis indicated that *PfrFAD3* only expressed in seed, not in leaf, stem and root tissues of perilla [13]. Since we are using qPCR which is more sensitive than Northern, we detected *PfrFAD3* expressed in leaf at a significant level similar to that of *PfrFAD*2 (Fig. 6d). In contrast, expression of *PfrFAD7/8* was not detected during the most stages of seed development (2–4 WAF), only a low level of the transcript was detected in young seeds at 1 WAF (Fig. 6d) where no seed TAG was measured. However, we found that *PfrFAD7/8* was highly expressed in leaf, indicating the importance of its function in leaf. Perilla contains high level of 18:3 FAs not only in seeds (Fig. 1c) but also in leaf (Additional file 1: Table S1). In leaves, glycerolipid (GL) moiety as PA or diacylglycerol (DAG) can flux between chloroplast and ER [31]. There are two linoleate desaturases, FAD7 and FAD8, structurally related with FAD3 in chloroplasts of Arabidopsis [47, 48]. Perilla leaf contains a high level of 18:3 up to 56.2 %, indicating that the majority of GL are transported from ER to chloroplast where GL-18:2 are then converted to GL-18:3 by FAD7/8 (Fig. 6a). Compared with PfrFAD3, PfrFAD7/8 N-terminus contains extra amino acid sequences encoding chloroplast transit peptide which supports the role of desaturation for membrane lipid in chloroplasts (Fig. 6b). Overall, our results of sequence and gene expression analyses provide essential information that PfrFAD3 and PfrFAD7/8 may be major enzymes for synthesizing 18:3 in seed TAGs and leaf membrane glycerolipids, respectively.

### TAG biosynthesis in ER

**Fig. 7:**
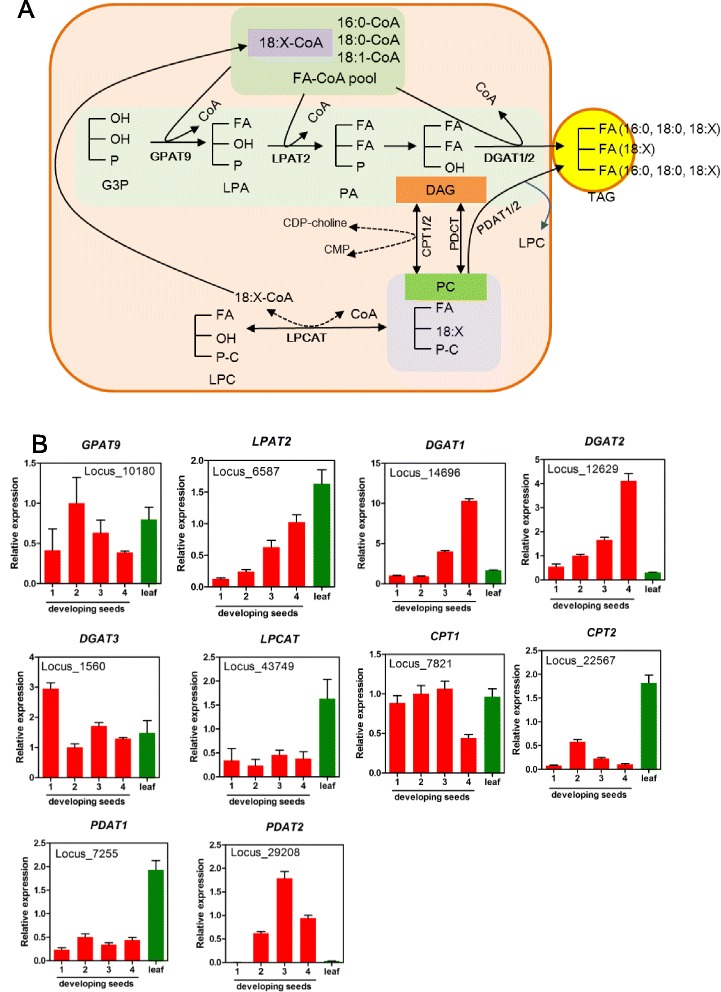
TAG synthesis and oil-body formation. **a** TAG biosynthesis through Kennedy pathway and PC-mediated pathway in seed. 18:X indicate unsaturated FA (18:1, 18:2 and 18:3). **b** Expression of genes involved in TAG biosynthesis. Abbreviations are described as in Fig. 2

Multiple mechanisms are involved in TAG biosynthesis in ER [25, 27, 28]. Acyl-CoAs in the cytosol can be incorporated into TAG through the glycerol-3-phosphate (G3P) pathway or the Kennedy pathway [49, 50], which involves three sequential acylation of acyl-CoAs into G3P. Firstly, G3P is acylated by glycerol-3-phosphate acyltransferase (GPAT), followed by a second acylation by the acyl-CoA:acylglycerol-3-phosphate acyltransferase (LPAT), yielding phosphatidic acid (PA). PA is then hydrolyzed to form diacylglycerol (DAG), which is finally used as a substrate for the diacylglycerol acyltranstransferase (DGAT) to produce TAG. The acyl-CoAs can also be incorporated directly into phospatidylcholine (PC) by the acyl editing reactions [25, 51, 52]. These acyl editing reactions can be catalyzed either by forward and reverse reactions of lyso-PC acyltransferase (LPCAT) to yield acyl-CoA, or by a phospholipase A–type activity to yield a free FA that then is activated to acyl-CoA. Since PC is the site for modified FA synthesis including 18:2, ALA, rapid de-acylation and re-acylation of PC results in an acyl-CoA pool enriched with unsaturated FAs which are then utilized for TAG synthesis [53, 54]. Besides, many plants utilize PC-mediated pathways to synthesize TAG. The enzyme Phospholipid:DAG acyltransferase (PDAT) syntheses TAG by transacylation of the *sn*-*2* FA from PC onto *sn-3* position of DAG [55]. FAs at the *sn*-1 and *sn*-2 position of PC in perilla can could be converted to TAG through DAG by phosphatidylcholine:diacylglycerol cholinephosphotransferase (PDCT) which exchanges phosphocholine between PC and DAG [56, 57]. CDP-choline:DAG cholinephosphotransferase (CPT) catalyzes the reaction of CDP-choline with DAG to generate PC. This reaction can be reversible [58–60]. PDCT and the reverse reaction of CPT would facilitate the FA on PC to be incorporated to TAG. A schematic drawing of TAG biosynthesis in perilla seeds is presented in Fig. 7a.

#### Genes involved in Kennedy pathway and acyl editing reactions

Based on the putative Arabidopsis GPAT9 (At5g60620) sequence [61], a perilla *GPAT9* (*PfrGPAT9*) transcript (Locus_10180) was found from the transcriptomes showing 81 % sequence identity to At5g60620 (Additional file 1: Table S8). *PfrGPAT9* transcript levels were comparable among leaf and developing seeds at different stages, although a bell-shaped pattern peaked at 2 WAF, the overall changes were about 2-fold or less (Fig. 7b). Perilla *LPAT2* (*PfrLPAT2*, Locus_6587), was identified using Arabidopsis LPAT2 (At3g57650) known to be involved in seed TAG biosynthesis [62]. Perilla and Arabidopsis LPAT2s share 81 % sequence identity. (Additional file 1: Table S8)*. PfrLPAT2* expression showed a continuous increase from 1 to 4 WAF during seed development, and its expression is higher in leave than seeds (Fig. 7b). The spatial and temporal expression patterns of perilla *GPAT9* and *LPAT2* suggest their constitutive functions with house-keeping roles in both membrane lipid and TAG synthesis. DGAT is the last enzyme in Kennedy pathway and often thought to be the rate limiting step in determining synthesis of TAG [63]. Perilla Locus_14696, Locus_12629 and Locus_1560 were revealed to encode *PfrDGAT1, PfrDGAT2,* and *PfrDGAT3* and showed 79, 67 and 42 % sequence identity with Arabidopsis *DGAT1* (At2g19450), *DGAT2* (At3g51520) and *DGAT3* (At1g48300), respectively, (Additional file 1: Table S8). *PfrDGAT1* and *PfrDGAT2* were expressed predominantly in seed, whereas *DGAT3* expressed both in seeds and leaf at similar levels (Fig. 7b). PfrDGAT1 and PfrDGAT2 are probably involved in TAG biosynthesis in seeds, whereas PfrDGAT3 is a house-keeping enzyme.

As polyunsaturated FAs (PUFA) are major components in TAG of perilla seeds, the acyl editing mechanism [28, 64] would enrich acyl-CoA pool with PUFA-CoAs, facilitating the incorporation of PUFAs into TAGs. Although there are two Arabidopsis *LPCATs, (LPCAT1*, At1g12640 and *LPCAT2*, At1g63050) were reported [64, 65], we found only one perilla LPCAT (Locus_43749) in transcriptomes and it expressed both in leaf and developing seeds (Fig. 7b). The finding of PfrLPCAT would suggest acyl-editing through PfrLPCAT likely utilized in perilla.

#### Genes involved in PC-mediated pathways for TAG biosysnthesis

TAG can be synthesized directly between DAG and PC by Phospholipid:diacylglycerol acyltransferase (PDAT) through acyl-CoA independent pathway [28, 64]. PDAT transfers FA of *sn*-*2* position in PC to *sn*-*3* position of DAG and synthesize TAG (Fig. 7a) [55, 66]. This mechanism has been demonstrated well with a castor PDAT. Castor seed oil contains 90 % ricinoleic acid (18:1OH) which is synthesized on the *sn*-2 of PC [58, 67]. When a castor *PDAT* (*RcPDAT*) was introduced into Arabidopsis expressing a castor fatty acid hydroxylase gene (*RcFAH12,* [59, 60]), the transgenic Arabidopsis with dual *RcFHA12* and *RcPDAT* enhanced 18:1OH level in TAG [68, 69]. Two perilla PDAT orthologs, PfrPDAT1 (Locus_7255) and PfrPDAT2 (Locus_29208), corresponding to Arabidopsis PDAT1 and PDAT2, respectively, were detected. *PfrPDAT1* expressed in seeds and leaves, whereas *PfrPDAT2* shows seed-specific expression (Fig. 7b). The spatial and temporal expression profiles of *PfrPDATs* are similar to that of Arabidopsis *PDATs* [66]. Our data of PfrPDATs provide molecular basis for further investigation of the role of PfrPDATs in ALA-containing TAG synthesis.

**Fig. 8:**
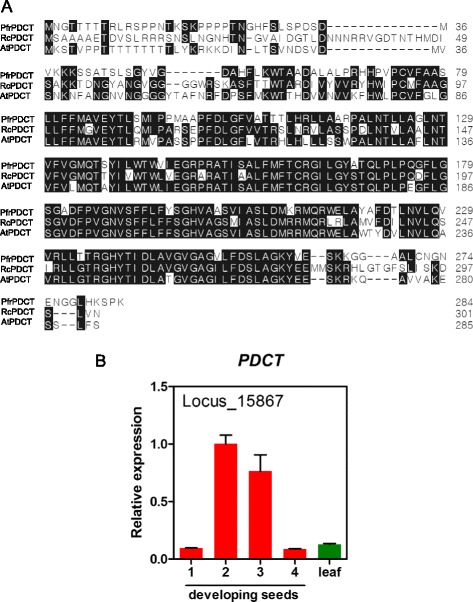
Characterization of perilla PDCT (phosphatidylcholine:diacylglycerol cholinephosphotransferase). **a** Amino acid sequence alignment of PfrPDCT, RcPDCT (Genbank accession XP_002517643) and AtPDCT (AT3G15820). **b** Expression of *PfrPDCT*

Perilla seed oil contains 60 % ALA (18:3) distributed at all three *sn*-1, 2, 3 positions of TAG, with somewhat higher at the *sn*-2 position [68]. However, perilla LPAT2 showed no activity in acylating ALA to the *sn*-2 position of TAG [68]. This indicates that the majority of ALA in the *sn*-2 TAG could be formed through PC-mediated DAG pathways, such as PDCT and reverse reaction of CPT, rather than Kennedy pathway (Fig. 7). We identified a full-length perilla PDCT (*PfrPDCT*, Locus_15867) cDNA showing 62 and 64 % identity with PDCTs from Arabidopsis and castor (Fig. 8a). *PfrPDCT* had a bell-shaped temporal expression pattern during seed development and also expressed significantly in leaf (Fig. 8b). The role of Arabidopsis PDCT (At3g15820) in contributing of unsaturated FA in TAGs has been demonstrated [57]. The castor PDCT has also shown to evolve to effectively convert 18:1OH-PC to 18:1OH-DAG for 18:1OH-containg TAG synthesis in transgenic Arabidopsis [56]. Besides, we have also identified two perilla CPT orthologs, CPT1 (PfrCPT1, Locus_7821) and CTP2 (PfrCPT2, Locus_22567) based on Arabidopsis AAPT1 (At1g13560) and AAPT2 (At3g25585). Both *PfrCPT1* and *PfrCPT2* expressed in seeds and leaves, but *PfrCPT2* had a relative higher level in leaves than seeds (Fig. 8b). The identification of *PfrPDCT* and *PfrCPTs* in this study helps to explain the ALAs at the *sn-2* of perilla TAGs that are likely acquired by PC-mediated DAG formation through *PfrPDCT* and *PfrCPTs*.

#### Oil body protein, oleosins

**Fig. 9:**
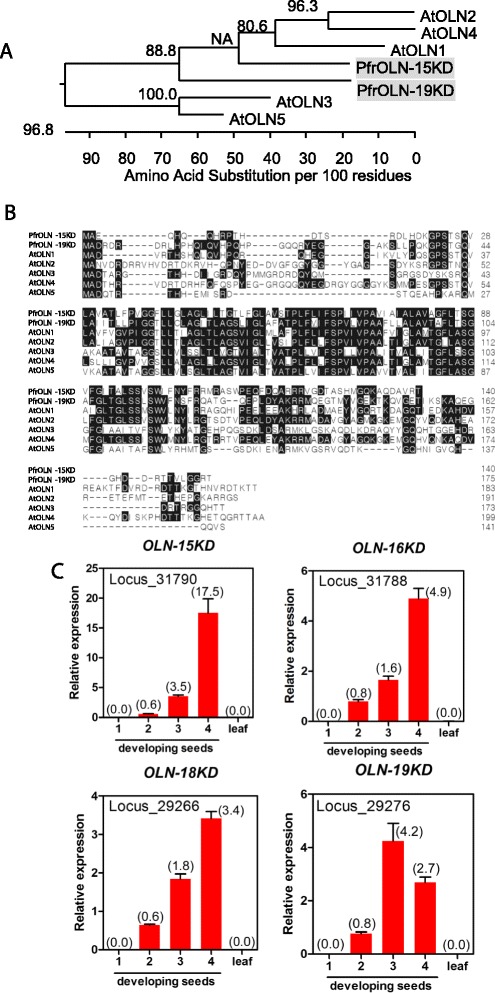
Characterization of perilla oleosins. **a** Phylogentic tree of perilla and Arabidopsis oleosins (OLN1 to OLN5 is AT3G01570, AT3G27660, AT4G25140, AT5G40420, AT5G51210 respectively) **b** Amino acid sequence alignment of perilla and Arabidopsis oleosins **c** Expression of genes encoding four perilla oleosins

TAGs is covered by single layer of phospholipids of ER and amphipathic oleosin (OLN) proteins. Arabidopsis encodes five seed-specific oleosin genes in the genome [70]. We have identified four different isoforms of oleosin from perilla seed transcriptome with molecular weight of 15, 16, 18, 19 KD (PfrOLN-15, −16, −18 and −19). Full-length of *PfrOLN-15* (AF210697.1) and PfrOLN-19 (AF237625.1) isoforms are closer to Arabidopsis OLN1 (AT3G01570) and they share a conserved harpin domain (~72 hydrophobic or neutral residues) with other olesions (Fig. 9a and b). All four *PfrOLNs* showed a seed-specific expression (Fig. 9c). However, the expression of *PfrOLN-15* had a linear-rise pattern showing a maximum 673.9-fold induction at 4 WFA compared with that of 1 WAF. The other *PfrOLNs* had much less dynamic changes showing either linear-rise (*PfrOLN-16, −18*) or bell-shaped (*PfrOLN-19*) patterns with a maximum induction between 103.4- and 188.5-fold (Fig. 9c). *PfrOLN-15* may be the major oleosin isoform in oil-body of perilla seeds (Fig. 9c).

In general, the expression profiles of genes involved in fatty acid and TAG biosynthesis detected by RNAseq analysis (Additional file 1: Table S8) and qPCR (Figs. 3, 4, 5, 6, 7, 8 and 9) are comparable, except for oleison genes. *PfrOLN-15* showed a highest expression in developing seeds using qPCR whereas *PfrOLN-19* and *PfrOLN-19* were highest using RNAseq. The discrepancy of expression level between RNAseq data and qPCR data was likely caused by chimeric transcripts generated by assemble program, which is inevitable in a assemble process purely based on de novo transcriptome data.

## Conclusions

*Perilla frutescens* (L.) var*. frutescens*, a valuable oilseed crop, contains high amount of ALA in seeds and leaves. Deep sequencing of cDNAs from developing perilla seeds and leaves was carried out to identify genes involved in the synthesis of seed TAG enriched with ALA. A total of 54,079 unique genes from 392 mega-base raw sequences were assembled. The majority (66 %, 21,429 out of 32,237) of the matched genes showed highest homology to *Mimulus guttatus* genes, confirming the close relationship between the two species. Genes involved in the synthesis of FA and TAG were identified and annotated by detailed sequence alignments. We have identified nearly all of the known genes for *de novo* FA biosynthesis in plastid, export from the plastid and TAG assembly in ER. In addition, we characterized the expression profiles of 43 key genes in TAG metabolism using quantitative PCR (qPCR). Two ω-3 fatty acid desaturase genes, *PfrFAD3* and *PfrFAD7/8* were identified as key genes for ALA synthesis in seeds and leaves, respectively. The identification of *PfrDGATs*, *PfrPDAT*s, *PfrPDCT* and *PfrCPTs* provides additional key genes not only for future studies on the mechanisms of ALA-containing TAG synthesis in perilla, but also for use as targets in genetic engineering of other oilseeds to produce a high level of ALA.

## Methods

### Plant materials and RNA extraction

Seeds of *Perilla frutescens* (L.) var *frutescens* cultivar ‘Dayudeulkkae’ were obtained from the National Institute of Crop Science, Miryang Republic of Korea. Perilla plants were grown in the greenhouse at temperatures between 18 and 28 °C. After fertilization, developing seeds from 1, 2, 3, 4 weeks and mature leaves were collected, immediately frozen in liquid nitrogen and stored at −80 °C prior to RNA extraction. Total RNAs from developing seeds and leaves of three replicates were extracted using the Plant RNA Reagent (Invitrogen) and treated with DNase I (Takara) according to manufacturer’s instructions. RNA quality was examined using 1 % agarose gel and the concentration was determined using a Nanodrap spectrophotometer (Thermo). The RNA integrity number determined by Agilent 2100 Bioanalyzer was greater than 7.0 for all RNA samples to construct cDNA libraries.

### Fatty acid content analysis

The fatty acid content of seeds and leaves were analyzed by gas chromatographic analysis with a known amount of 15:0 fatty acid as an internal standard. Samples were transmethylated at 90 °C for 90 min in 0.3 mL of toluene and 1 mL of 5 % H_2_SO_4_ (v/v methanol). After transmethylation, 1,5 mL of 0.9 % NaCl solution was added, and the fatty acid methyl esters (FAMEs) were transferred to a new tube for three sequential extraction with 1.5 mL of n-hexane. FAMEs were analyzed by gas chromatography using a GC-2010 plus instrument (Shimadzu, Japan) with 1 30 m × 0.25 um (inner diameter) HP-FFAP column (Agilent, USA), during which the oven temperature was increased from 170 to 180 °C at 1 °C/min.

### cDNA library construction and massive parallel sequencing

RNA-Seq paired end libraries were prepared using the Illumina TruSeq RNA Sample Preparation Kit v2 (catalog #RS-122-2001, Illumina, San Diego, CA). Based on the instruction provided by the kit, mRNAs were purified from total RNA using poly (A) selection, and then chemically fragmented and converted into single-stranded cDNA. Using random hexamer priming, a second strand is generated to create double-stranded (ds) cDNAs. Library construction begins with generation of blunt-end cDNA fragments from ds-cDNAs. Then Adenine nucleotide (A)-base added to the blunt-end in order to make them ready for ligation of sequencing adapters. After the size selection of ligates, the ligated cDNA fragments which contain adapter sequences are enhanced via PCR using adapter specific primers. The library was quantified with KAPA library quantification kit (Kapa biosystems KK4854) following the manufacturer’s instructions. Each library was sequenced using Illumina Hiseq2000 platform, which created 100 bp paired-end sequencing reads.

### *De novo* assembly and unique transcripts annotation

Raw sequencing data composed of 100 bp paired-end reads filtered by Phred quality score (Q ≥ 20) and read length (≥25 bp) with SolexaQA [71]. We used all the sequence reads from different tissue samples to optimize the *de novo* assembly using the software tools Velvet (v1.2.07) [72] to assess *k*-mer sizes and assembled contigs. The contigs were joined into transcript isoforms using Oases (v0.2.08) [73]. Velvet and Oases are based on the de Bruijn graph algorithm. We took several hash length into consideration to select the best de novo assembly. The unique transcripts of perilla were defined by merging the best *de novo* assembly and validated by direct comparison with gene sequences in the Phytozome (http://www.phytozome.net/) using BLASTx (e-value ≤ 1E-10). The proteins with the highest sequence similarity were retrieved for analysis.

### Short read mapping and expression profiles in experimental samples

Reads for each sequence tag were mapped to the assembled unique transcripts using Bowtie software (v2.10) [19]. The number of mapped clean reads for each unique transcript was counted and then normalized with DESeq package in [74]. Only those representative transcripts with mapped reads counts of 1000 or above in at least one experimental sample were retained for further analysis. Fold change and binomial-Test were used to identify differentially expressed genes between each sample. FDR (false discovery rate) was applied to identify the threshold of the *p*-value in multiple tests and analysis and this value was calculated via DESeq. All correlation analysis, hierarchical clustering was performed using AMAP library in R [20].

### GO analysis

Gene Ontology (GO) analysis was carried out via DAVID (http://david.abcc.ncifcrf.gov/tools.jsp) [24]. The gene lists by annotated TAIR ID of transcripts of up- and down-regulated DEG were analyzed with counts ≥ 5 and FDR ≤ 0.01 of each GO terms.

### Quantitative PCR

Total RNA were reverse transcribed with the PrimeScrip™ 1st strand cDNA synthesis kit (Takara, Japan) according to manufacturer’s protocol. Real-time PCR was performed using the SYBR® Premix Ex Taq™ II (Takara, Japan) on the CFX96 Real-Time PCR system (Bio-Rad) with gene-specific primer pairs (Additional file 1: Table S5). Perilla *ACTIN* (AB002819.1) was used as the internal reference gene. The relative expression value was calculated via the ΔΔCt method.

### Full-length cDNA cloning and sequence analysis

A cDNA containing full-length open reading frame (ORF) for *FAD2. FAD3* and *PDCT* were amplified using KOD polymerase from total RNA of developing seeds or leaves samples using primers (Additional file 1: Table S9). PCR products were cloned into pCR-Blunt vector (Invitrogen) for Sanger sequencing. The amino acid sequence alignment of proteins was performed with CLUSTALW program of DNASTAR software with default parameters. Phylogenetic tree was built with the CLUSTALW method with DNASTAR MegAlign program.

## Additional files

Additional file 1:
**Table S1.** Fatty acid composition of perilla developing seeds and leaves. All data are averages of three measurements ± SE. **Table S2.** Statistical summary of *de novo* transcriptome assembly and annotation. **Table S3.** Annotation of 54,079 perilla representative transcripts and their expression in developing seeds and leaf. Transcripts represents as Locus. Raw read count and normalized read count were represented as expression level for each developing seeds and leaves samples. **Table S4.** Nucleotide sequences of 54,079 perilla representative transcripts in FASTA format. **Table S5.** Gene ontology analysis of DEG cluster 3, 4, 5 and 10 transcripts of perilla. **Table S6.** List of gene products for the top 50 DEGs in Cluster 3, 4, 5 and 10. **Table S7.** Number of identified genes involved in acyl-lipid metabolism of perilla transcriptome. **Table S8.** List of expressed genes involved in fatty acid and TAG biosynthesis in perilla. **Table S9.** Information of perilla gene primers (5′ → 3′) used in the qPCR analysis and cDNA cloning. (ZIP 27440 kb) Additional file 2:
**Figure S1.** Sequence length distribution of transcripts. Data represent the assembled transcripts (red bar) and unique transcripts (green bar) from both leaf and seeds. **Figure S2.** Annotated profile in Phytozome databases of 32,237 perilla unique transcripts. Perilla transcripts were searched with BlastX algorithm. **Figure S3.** Changes in gene expression during seed development. Numbers of up- (red bar) or down-regulated (green bar) genes in developing seeds of 1–4 weeks after flowering were determined by analysis of differentially expression genes using leaf sample as a control. **Figure S4.** Hierarchical clustering analysis of 6012 DEGs based on log ratio RPKM data. (A) Heatmap. (B) Line plot for 12 clusters. Fold changes of DEGs in developing seeds (1–4 week after flowering, WAF) are calculated based on leaf value. **Figure S5.** Characterization of perilla FAD2. (A) Amino acid sequence alignment of perilla and Arabidopsis FAD2. Red boxes indicate His conserved motifs. (B) Phylogentic tree of plant FAD2s. Abbreviations: Ah*, Arachis hypogaea*; At*, Arabidopsis thaliana*; Bc*, Brassica carinata*; Bj*, Brassica juncea*; Br*, Brassica rapa*; Cs*, Camelina sativa*; Ct*, Carthamus tinctorius*; El*, Euphorbia lagascae*; Gm*, Glycine max*; Gh*, Gossypium hirsutum*; Ha*, Helianthus annuus*; Jc*, Jatropha curcus*; Lu*, Linum usitatissimum*; Oe*, Olea europaea*; Pfr, *Perilla frutescens* Rc*, Ricinus communis*; Si*, Sesamum indicum*; So*, Spinacia oleracea*; Vf*, Vernicia fordii*; Vl*, Vitis labrusca.* **Figure S6.** Perilla FAD3 and FAD7/8 amino acid sequence alignment with Arabidopsis FAD3, FAD7 and FAD8. Red box indicate His conserved motifs. Abbreviations are described in Additional file 2: Figure S5. AtFAD3 (AT2G29980), AtFAD7 (AT3G11170), AtFAD8 (AT5G05580). (PPTX 1366 kb)

## References

[1] Nitta M, Lee JK, Ohnishi O (2003). Asian perilla crops and their weedy forms: Their cultivation, utilization and genetic relationships. Econ Bot.

[2] Asif M (2011). Health effects of omega-3,6,9 fatty acids: *Perilla frutescens* is a good example of plant oils. Orient Pharm Exp Med.

[3] Simopoulos AP (1991). Omega-3 fatty acids in health and disease and in growth and development. Am J Clin Nutr.

[4] Wijendran V, Hayes KC (2004). Dietary n-6 and n-3 fatty acid balance and cardiovascular health. Annu Rev Nutr.

[5] Simopoulos AP (2000). Human requirement for N-3 polyunsaturated fatty acids. Poult Sci.

[6] Brenner DM. Perilla, Botany, Uses and Genetic Resources. J Janick and JE 1993.

[7] Müller-Waldeck F, Sitzmann J, Schnitzler WH, Graßmann J (2010). Determination of toxic perilla ketone, secondary plant metabolites and antioxidative capacity in five *Perilla frutescens* L. varieties. Food Chem Toxicol.

[8] Yang SY, Hong CO, Lee H, Park SY, Park BG, Lee KW (2012). Protective effect of extracts of *Perilla frutescens* treated with sucrose on tert-butyl hydroperoxide-induced oxidative hepatotoxicity in vitro and in vivo. Food Chem.

[9] Yamazaki M, Shibata M, Nishiyama Y, Springob K, Kitayama M, Shimada N, Aoki T, Ayabe S, Saito K (2008). Differential gene expression profiles of red and green forms of *Perilla frutescens* leading to comprehensive identification of anthocyanin biosynthetic genes. FEBS J.

[10] Mau CJ, Karp F, Ito M, Honda G, Croteau RB (2010). A candidate cDNA clone for (−)-limonene-7-hydroxylase from *Perilla frutescens*. Phytochemistry.

[11] Tong W, Kwon SJ, Lee J, Choi IY, Park YJ, Choi SH, Sa KJ, Kim BW, Lee JK (2015). Gene set by *de novo* assembly of Perilla species and expression profiling between *P. frutescens* (L.) var. frutescens and var. crispa. Gene.

[12] Fukushima A, Nakamura M, Suzuki H, Saito K, Yamazaki M (2015). High-Throughput Sequencing and *De Novo* Assembly of Red and Green Forms of the *Perilla frutescens* var. crispa Transcriptome. PLoS One.

[13] Chung CH, Kim JL, Lee YC, Choi YL (1999). Cloning and characterization of a seed-specific omega-3 fatty acid desaturase cDNA from *Perilla frutescens*. Plant Cell Physiol.

[14] Rao S, Abdel-Reheem M, Bhella R, McCracken C, Hildebrand D (2008). Characteristics of high alpha-linolenic acid accumulation in seed oils. Lipids.

[15] Chung KJ, Hwang SK, Hahn BS, Kim KH, Kim JB, Kim YH, Yang JS, Ha SH (2008). Authentic seed-specific activity of the perilla oleosin 19 gene promoter in transgenic Arabidopsis. Plant Cell Rep.

[16] Sreedhar RV, Kumari P, Rupwate SD, Rajasekharan R, Srinivasan M (2015). Exploring triacylglycerol biosynthetic pathway in developing seeds of Chia (Salvia hispanica L.): a transcriptomic approach. PLoS One.

[17] Wang X, Xu R, Wang R, Liu A (2012). Transcriptome analysis of Sacha Inchi (Plukenetia volubilis L) seeds at two developmental stages. BMC Genomics.

[18] Hellsten U, Wright KM, Jenkins J, Shu S, Yuan Y, Wessler SR, Schmutz J, Willis JH, Rokhsar DS (2013). Fine-scale variation in meiotic recombination in Mimulus inferred from population shotgun sequencing. Proc Natl Acad Sci U S A.

[19] Langmead B, Trapnell C, Pop M, Salzberg SL (2009). Ultrafast and memory-efficient alignment of short DNA sequences to the human genome. Genome Biol.

[20] Lucas A (2014). amap: Another Multidimensional Analysis Package. R package version 0.8–12. ed.

[21] Wang X, Liu A (2014). Expression of genes controlling unsaturated fatty acids biosynthesis and oil deposition in developing seeds of Sacha inchi (*Plukenetia volubilis* L.). Lipids.

[22] Zhang Y, Peng L, Wu Y, Shen Y, Wu X, Wang J (2014). Analysis of global gene expression profiles to identify differentially expressed genes critical for embryo development in *Brassica rapa*. Plant Mol Biol.

[23] Jiang H, Wu P, Zhang S, Song C, Chen Y, Li M, Jia Y, Fang X, Chen F, Wu G (2012). Global analysis of gene expression profiles in developing physic nut (*Jatropha curcas* L.) seeds. PLoS One.

[24] da Huang W, Sherman BT, Lempicki RA (2009). Systematic and integrative analysis of large gene lists using DAVID bioinformatics resources. Nat Protoc.

[25] Li-Beisson Y, Shorrosh B, Beisson F, Andersson MX, Arondel V, Bates PD, Baud S, Bird D, Debono A, Durrett TP (2013). Acyl-lipid metabolism. Arabidopsis Book.

[26] Kim HU, Chen GQ (2015). Identification of hydroxy fatty acid and triacylglycerol metabolism-related genes in lesquerella through seed transcriptome analysis. BMC Genomics.

[27] Chapman KD, Ohlrogge JB (2012). Compartmentation of triacylglycerol accumulation in plants. J Biol Chem.

[28] Bates PD, Stymne S, Ohlrogge J (2013). Biochemical pathways in seed oil synthesis. Curr Opin Plant Biol.

[29] Johnston ML, Luethy MH, Miernyk JA, Randall DD (1997). Cloning and molecular analyses of the Arabidopsis thaliana plastid pyruvate dehydrogenase subunits. Biochim Biophys Acta.

[30] Konishi T, Shinohara K, Yamada K, Sasaki Y (1996). Acetyl-CoA carboxylase in higher plants: most plants other than gramineae have both the prokaryotic and the eukaryotic forms of this enzyme. Plant Cell Physiol.

[31] Ohlrogge J, Browse J (1995). Lipid biosynthesis. Plant Cell.

[32] Brown AP, Affleck V, Fawcett T, Slabas AR (2006). Tandem affinity purification tagging of fatty acid biosynthetic enzymes in Synechocystis sp. PCC6803 and *Arabidopsis thaliana*. J Exp Bot.

[33] To A, Joubes J, Barthole G, Lecureuil A, Scagnelli A, Jasinski S, Lepiniec L, Baud S (2012). WRINKLED transcription factors orchestrate tissue-specific regulation of fatty acid biosynthesis in Arabidopsis. Plant Cell.

[34] Shen B, Allen WB, Zheng P, Li C, Glassman K, Ranch J, Nubel D, Tarczynski MC (2010). Expression of ZmLEC1 and ZmWRI1 increases seed oil production in maize. Plant Physiol.

[35] Liu J, Hua W, Zhan G, Wei F, Wang X, Liu G, Wang H (2010). Increasing seed mass and oil content in transgenic Arabidopsis by the overexpression of wri1-like gene from *Brassica napus*. Plant Physiol Biochem.

[36] Kachroo A, Shanklin J, Whittle E, Lapchyk L, Hildebrand D, Kachroo P (2007). The Arabidopsis stearoyl-acyl carrier protein-desaturase family and the contribution of leaf isoforms to oleic acid synthesis. Plant Mol Biol.

[37] Dussert S, Guerin C, Andersson M, Joet T, Tranbarger TJ, Pizot M, Sarah G, Omore A, Durand-Gasselin T, Morcillo F (2013). Comparative transcriptome analysis of three oil palm fruit and seed tissues that differ in oil content and fatty acid composition. Plant Physiol.

[38] Schnurr JA, Shockey JM, de Boer GJ, Browse JA (2002). Fatty acid export from the chloroplast. Molecular characterization of a major plastidial acyl-coenzyme A synthetase from Arabidopsis. Plant Physiol.

[39] Zhao L, Katavic V, Li F, Haughn GW, Kunst L (2010). Insertional mutant analysis reveals that long-chain acyl-CoA synthetase 1 (LACS1), but not LACS8, functionally overlaps with LACS9 in Arabidopsis seed oil biosynthesis. Plant J.

[40] Sperling P, Heinz E (1993). Isomeric sn-1-octadecenyl and sn-2-octadecenyl analogues of lysophosphatidylcholine as substrates for acylation and desaturation by plant microsomal membranes. Eur J Biochem.

[41] Okuley J, Lightner J, Feldmann K, Yadav N, Lark E, Browse J (1994). Arabidopsis FAD2 gene encodes the enzyme that is essential for polyunsaturated lipid synthesis. Plant Cell.

[42] Browse J, McConn M, James D, Miquel M (1993). Mutants of Arabidopsis deficient in the synthesis of alpha-linolenate. Biochemical and genetic characterization of the endoplasmic reticulum linoleoyl desaturase. J Biol Chem.

[43] Lee KR, In Sohn S, Jung JH, Kim SH, Roh KH, Kim JB, Suh MC, Kim HU (2013). Functional analysis and tissue-differential expression of four FAD2 genes in amphidiploid *Brassica napus* derived from *Brassica rapa* and *Brassica oleracea*. Gene.

[44] Kang J, Snapp AR, Lu C (2011). Identification of three genes encoding microsomal oleate desaturases (FAD2) from the oilseed crop *Camelina sativa*. Plant Physiol Biochem.

[45] Yurchenko OP, Park S, Ilut DC, Inmon JJ, Millhollon JC, Liechty Z, Page JT, Jenks MA, Chapman KD, Udall JA (2014). Genome-wide analysis of the omega-3 fatty acid desaturase gene family in Gossypium. BMC Plant Biol.

[46] Khadake R, Khonde V, Mhaske V, Ranjekar P, Harsulkar A (2011). Functional and bioinformatic characterisation of sequence variants of Fad3 gene from flax. J Sci Food Agric.

[47] Gibson S, Arondel V, Iba K, Somerville C (1994). Cloning of a temperature-regulated gene encoding a chloroplast omega-3 desaturase from *Arabidopsis thaliana*. Plant Physiol.

[48] McConn M, Hugly S, Browse J, Somerville C (1994). A Mutation at the fad8 Locus of Arabidopsis Identifies a Second Chloroplast [omega]-3 Desaturase. Plant Physiol.

[49] Weiss SB, Kennedy EP, Kiyasu JY (1960). The enzymatic synthesis of triglycerides. J Biol Chem.

[50] Weiss SB, Kennedy EP (1956). The enzymatic synthesis of triglycerides. J Am Chem Soc.

[51] Lands WE (1965). Lipid Metabolism. Annu Rev Biochem.

[52] Bates PD, Browse J (2012). The significance of different diacylgycerol synthesis pathways on plant oil composition and bioengineering. Front Plant Sci.

[53] Stymne S, Stobart AK (1984). Evidence for the reversibility of the acyl-CoA:lysophosphatidylcholine acyltransferase in microsomal preparations from developing safflower (*Carthamus tinctorius* L.) cotyledons and rat liver. Biochem J.

[54] Stahl U, Stalberg K, Stymne S, Ronne H (2008). A family of eukaryotic lysophospholipid acyltransferases with broad specificity. FEBS Lett.

[55] Dahlqvist A, Stahl U, Lenman M, Banas A, Lee M, Sandager L, Ronne H, Stymne S (2000). Phospholipid:diacylglycerol acyltransferase: an enzyme that catalyzes the acyl-CoA-independent formation of triacylglycerol in yeast and plants. Proc Natl Acad Sci U S A.

[56] Hu Z, Ren Z, Lu C (2012). The phosphatidylcholine diacylglycerol cholinephosphotransferase is required for efficient hydroxy fatty acid accumulation in transgenic Arabidopsis. Plant Physiol.

[57] Lu C, Xin Z, Ren Z, Miquel M, Browse J (2009). An enzyme regulating triacylglycerol composition is encoded by the ROD1 gene of Arabidopsis. Proc Natl Acad Sci U S A.

[58] Bafor M, Smith MA, Jonsson L, Stobart K, Stymne S (1991). Ricinoleic Acid Biosynthesis and Triacylglycerol Assembly in Microsomal Preparations from Developing Castor-Bean (*Ricinus communis*) Endosperm. Biochem J.

[59] van de Loo FJ, Broun P, Turner S, Somerville C (1995). An oleate 12-hydroxylase from Ricinus communis L. is a fatty acyl desaturase homolog. Proc Natl Acad Sci U S A.

[60] Lu C, Fulda M, Wallis JG, Browse J (2006). A high-throughput screen for genes from castor that boost hydroxy fatty acid accumulation in seed oils of transgenic Arabidopsis. Plant J.

[61] Gidda SK, Shockey JM, Rothstein SJ, Dyer JM, Mullen RT (2009). *Arabidopsis thaliana* GPAT8 and GPAT9 are localized to the ER and possess distinct ER retrieval signals: functional divergence of the dilysine ER retrieval motif in plant cells. Plant Physiol Biochem.

[62] Kim HU, Li Y, Huang AH (2005). Ubiquitous and endoplasmic reticulum-located lysophosphatidyl acyltransferase, LPAT2, is essential for female but not male gametophyte development in Arabidopsis. Plant Cell.

[63] Liu Q, Siloto RM, Lehner R, Stone SJ, Weselake RJ (2012). Acyl-CoA:diacylglycerol acyltransferase: molecular biology, biochemistry and biotechnology. Prog Lipid Res.

[64] Xu J, Carlsson AS, Francis T, Zhang M, Hoffman T, Giblin ME, Taylor DC (2012). Triacylglycerol synthesis by PDAT1 in the absence of DGAT1 activity is dependent on re-acylation of LPC by LPCAT2. BMC Plant Biol.

[65] Bates PD, Fatihi A, Snapp AR, Carlsson AS, Browse J, Lu C (2012). Acyl editing and headgroup exchange are the major mechanisms that direct polyunsaturated fatty acid flux into triacylglycerols. Plant Physiol.

[66] Stahl U, Carlsson AS, Lenman M, Dahlqvist A, Huang B, Banas W, Banas A, Stymne S (2004). Cloning and functional characterization of a phospholipid:diacylglycerol acyltransferase from Arabidopsis. Plant Physiol.

[67] Galliard T, Stumpf PK (1966). Fat metabolism in higher plants. 30. Enzymatic synthesis of ricinoleic acid by a microsomal preparation from developing *Ricinus communis* seeds. J Biol Chem.

[68] Kim HU, Lee KR, Go YS, Jung JH, Suh MC, Kim JB (2011). Endoplasmic reticulum-located PDAT1-2 from castor bean enhances hydroxy fatty acid accumulation in transgenic plants. Plant Cell Physiol.

[69] van Erp H, Bates PD, Burgal J, Shockey J, Browse J (2011). Castor phospholipid:diacylglycerol acyltransferase facilitates efficient metabolism of hydroxy fatty acids in transgenic Arabidopsis. Plant Physiol.

[70] Kim HU, Hsieh K, Ratnayake C, Huang AH (2002). A novel group of oleosins is present inside the pollen of Arabidopsis. J Biol Chem.

[71] Cox MP, Peterson DA, Biggs PJ (2010). SolexaQA: At-a-glance quality assessment of Illumina second-generation sequencing data. BMC Bioinformatics.

[72] Zerbino DR, Birney E (2008). Velvet: algorithms for *de novo* short read assembly using de Bruijn graphs. Genome Res.

[73] Schulz MH, Zerbino DR, Vingron M, Birney E (2012). Oases: robust *de novo* RNA-seq assembly across the dynamic range of expression levels. Bioinformatics.

[74] Anders S, Huber W (2010). Differential expression analysis for sequence count data. Genome Biol.

