# Supplementary material for: Transcriptome analysis and identification of genes associated with ω-3 fatty acid biosynthesis in Perilla frutescens (L.) var. frutescens
Source: BMC Genomics. 2016 Jun 24;17:474. doi: 10.1186/s12864-016-2805-0 (PMC4920993; doi:10.1186/s12864-016-2805-0)
Supplement: Additional file 2: — Figure S1. Sequence length distribution of transcripts. Data represent the assembled transcripts (red bar) and unique transcripts (green bar) from both leaf and seeds. Figure S2. Annotated profile in Phytozome databases of 32,237 perilla unique transcripts. Perilla transcripts were searched with BlastX algorithm. Figure S3. Changes in gene expression during seed development. Numbers of up- (red bar) or down-regulated (green bar) genes in developing seeds of 1–4 weeks after flowering were determined by analysis of differentially expression genes using leaf sample as a control. Figure S4. Hierarchical clustering analysis of 6012 DEGs based on log ratio RPKM data. (A) Heatmap. (B) Line plot for 12 clusters. Fold changes of DEGs in developing seeds (1–4 week after flowering, WAF) are calculated based on leaf value. Figure S5. Characterization of perilla FAD2. (A) Amino acid sequence alignment of perilla and Arabidopsis FAD2. Red boxes indicate His conserved motifs. (B) Phylogentic tree of plant FAD2s. Abbreviations: Ah, Arachis hypogaea; At, Arabidopsis thaliana; Bc, Brassica carinata; Bj, Brassica juncea; Br, Brassica rapa; Cs, Camelina sativa; Ct, Carthamus tinctorius; El, Euphorbia lagascae; Gm, Glycine max; Gh, Gossypium hirsutum; Ha, Helianthus annuus; Jc, Jatropha curcus; Lu, Linum usitatissimum; Oe, Olea europaea; Pfr, Perilla frutescens Rc, Ricinus communis; Si, Sesamum indicum; So, Spinacia oleracea; Vf, Vernicia fordii; Vl, Vitis labrusca. Figure S6. Perilla FAD3 and FAD7/8 amino acid sequence alignment with Arabidopsis FAD3, FAD7 and FAD8. Red box indicate His conserved motifs. Abbreviations are described in Additional file 2: Figure S5. AtFAD3 (AT2G29980), AtFAD7 (AT3G11170), AtFAD8 (AT5G05580). (PPTX 1366 kb) [file 12864_2016_2805_MOESM2_ESM.pptx]

## Slide 1
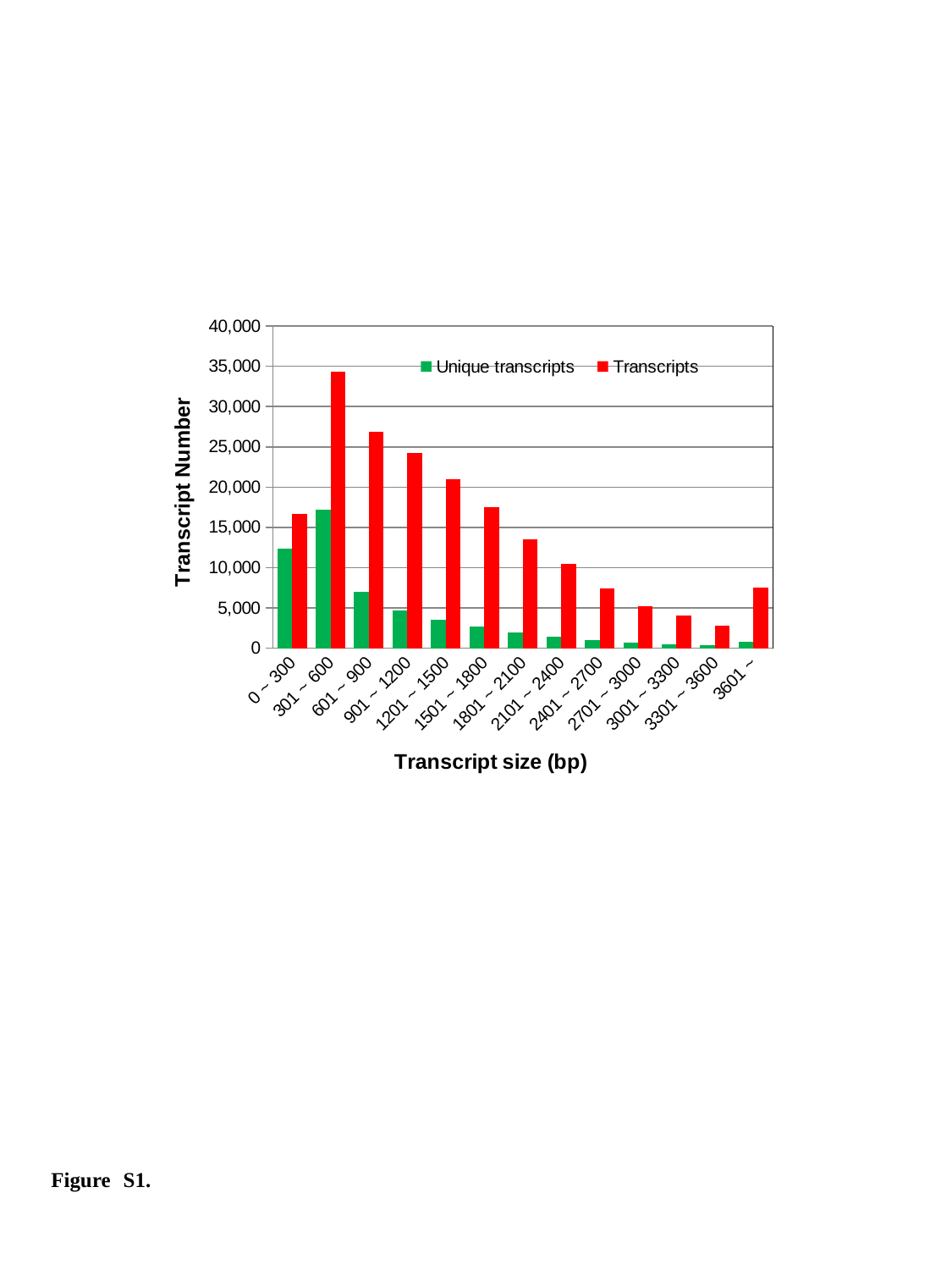

### Chart
| Category | Unique transcripts | Transcripts |
|---|---|---|
| 0 ~ 300 | 12346.0 | 16697.0 |
| 301 ~ 600 | 17192.0 | 34370.0 |
| 601 ~ 900 | 7012.0 | 26826.0 |
| 901 ~ 1200 | 4706.0 | 24279.0 |
| 1201 ~ 1500 | 3531.0 | 21017.0 |
| 1501 ~ 1800 | 2680.0 | 17514.0 |
| 1801 ~ 2100 | 1913.0 | 13473.0 |
| 2101 ~ 2400 | 1407.0 | 10479.0 |
| 2401 ~ 2700 | 991.0 | 7383.0 |
| 2701 ~ 3000 | 692.0 | 5205.0 |
| 3001 ~ 3300 | 498.0 | 4005.0 |
| 3301 ~ 3600 | 336.0 | 2808.0 |
| 3601 ~ | 775.0 | 7489.0 |Figure S1.

## Slide 2
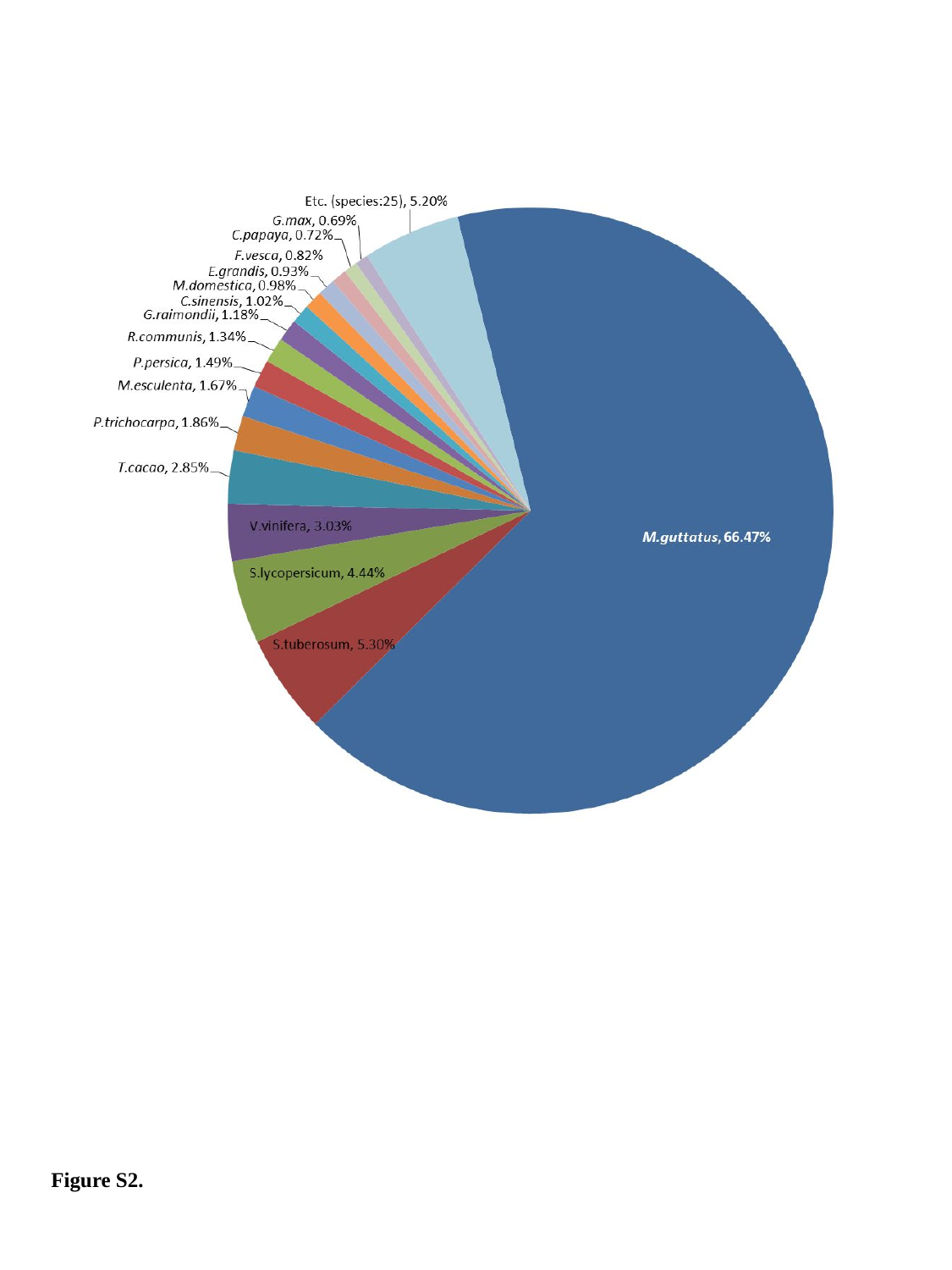

Figure S2.

## Slide 3
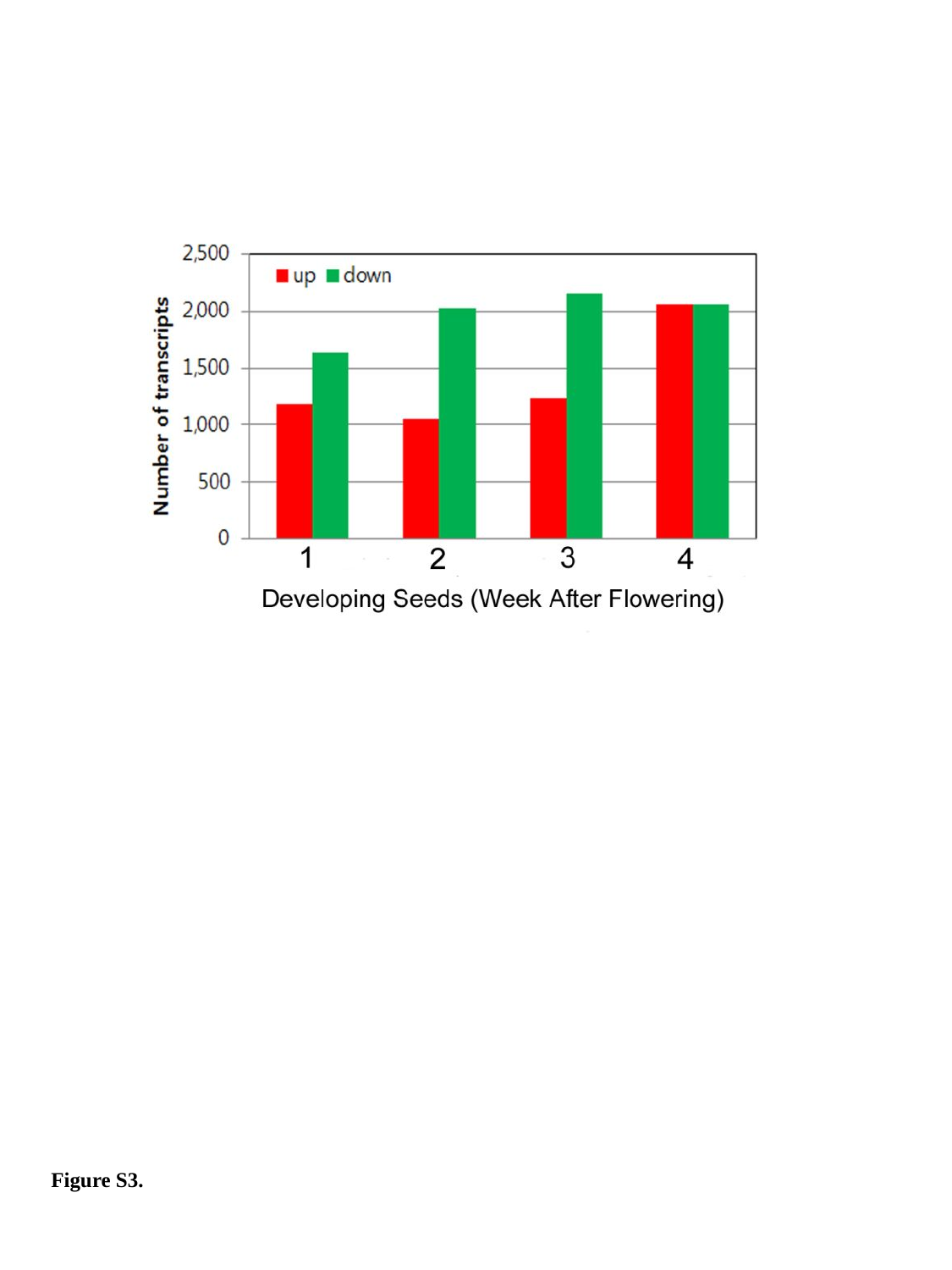

Figure S3.

## Slide 4
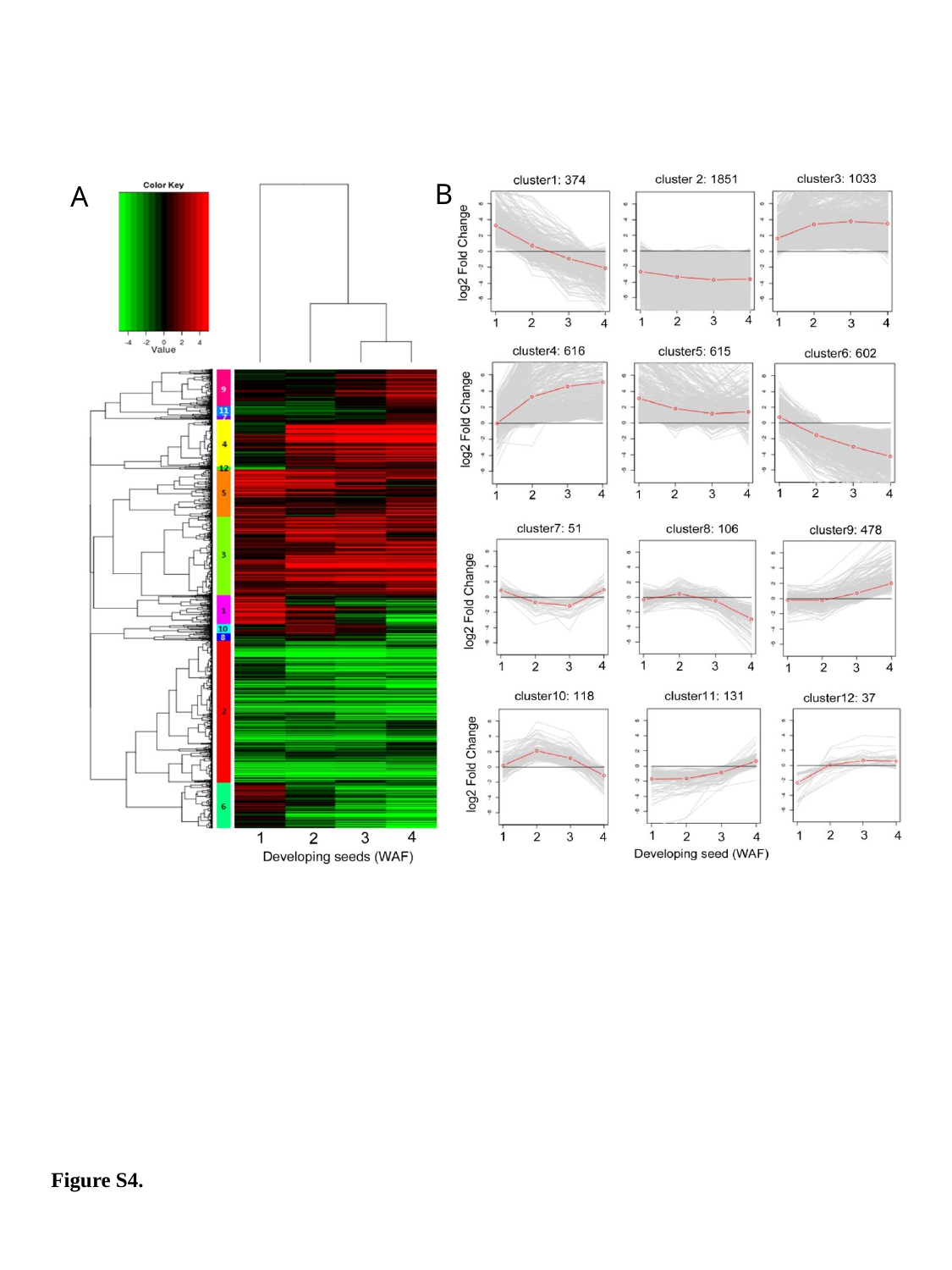

B
A
Figure S4.

## Slide 5
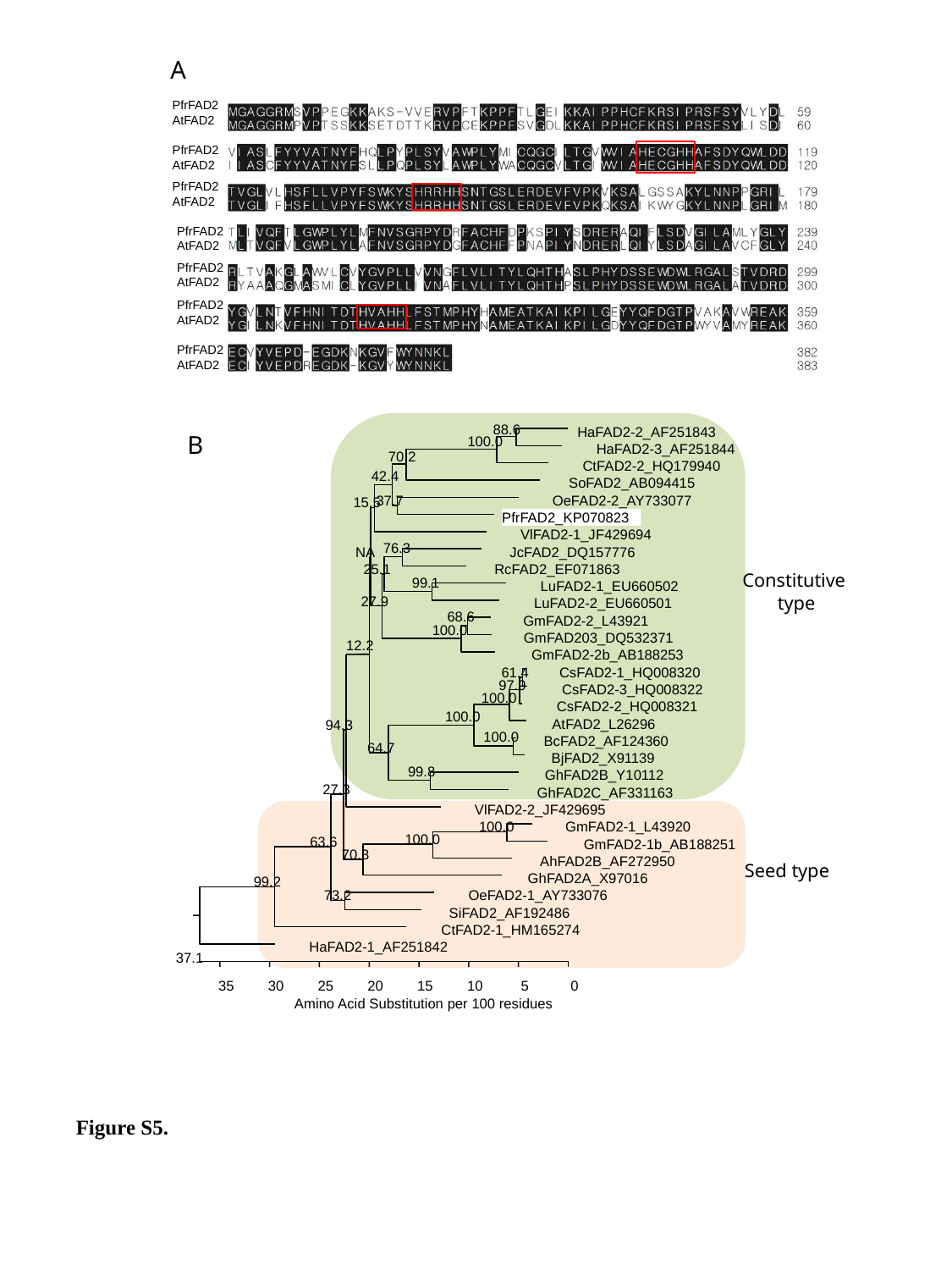

A
PfrFAD2
AtFAD2
PfrFAD2
AtFAD2
PfrFAD2
AtFAD2
PfrFAD2
AtFAD2
PfrFAD2
AtFAD2
PfrFAD2
AtFAD2
PfrFAD2
AtFAD2
88.6
HaFAD2-2_AF251843
100.0
HaFAD2-3_AF251844
70.2
CtFAD2-2_HQ179940
42.4
SoFAD2_AB094415
37.7
OeFAD2-2_AY733077
15.5
PfrFAD2_KP070823
VlFAD2-1_JF429694
76.3
NA
JcFAD2_DQ157776
25.1
RcFAD2_EF071863
99.1
LuFAD2-1_EU660502
27.9
LuFAD2-2_EU660501
68.6
GmFAD2-2_L43921
100.0
GmFAD203_DQ532371
12.2
GmFAD2-2b_AB188253
61.4
CsFAD2-1_HQ008320
97.9
CsFAD2-3_HQ008322
100.0
CsFAD2-2_HQ008321
100.0
AtFAD2_L26296
94.3
100.0
BcFAD2_AF124360
64.7
BjFAD2_X91139
99.8
GhFAD2B_Y10112
27.3
GhFAD2C_AF331163
VlFAD2-2_JF429695
100.0
GmFAD2-1_L43920
100.0
63.6
GmFAD2-1b_AB188251
70.3
AhFAD2B_AF272950
GhFAD2A_X97016
99.2
73.2
OeFAD2-1_AY733076
SiFAD2_AF192486
CtFAD2-1_HM165274
HaFAD2-1_AF251842
37.1
35
30
25
20
15
10
5
0
Amino Acid Substitution per 100 residues
B
Constitutive
type
Seed type
Figure S5.

## Slide 6
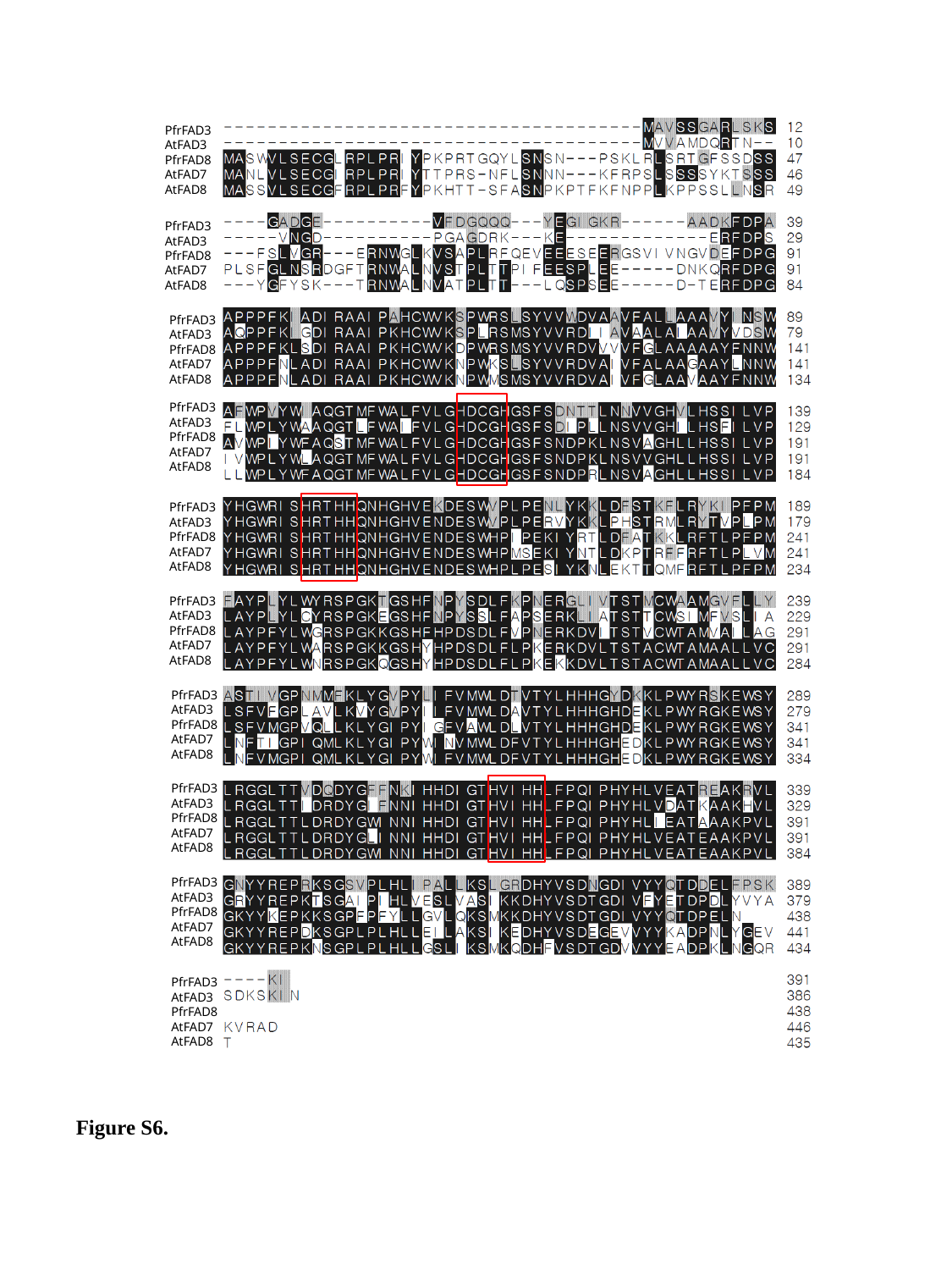

PfrFAD3
AtFAD3
PfrFAD8
AtFAD7
AtFAD8
PfrFAD3
AtFAD3
PfrFAD8
AtFAD7
AtFAD8
PfrFAD3
AtFAD3
PfrFAD8
AtFAD7
AtFAD8
PfrFAD3
AtFAD3
PfrFAD8
AtFAD7
AtFAD8
PfrFAD3
AtFAD3
PfrFAD8
AtFAD7
AtFAD8
PfrFAD3
AtFAD3
PfrFAD8
AtFAD7
AtFAD8
PfrFAD3
AtFAD3
PfrFAD8
AtFAD7
AtFAD8
PfrFAD3
AtFAD3
PfrFAD8
AtFAD7
AtFAD8
PfrFAD3
AtFAD3
PfrFAD8
AtFAD7
AtFAD8
PfrFAD3
AtFAD3
PfrFAD8
AtFAD7
AtFAD8
Figure S6.
